# Nanocatalytic system releases overloaded zinc ions and ROS to induce Znproptosis and interrupt cell cycle through inhibiting Akt/mTOR pathway

**DOI:** 10.7150/thno.107025

**Published:** 2025-03-24

**Authors:** Zi-Yue Xi, Chuan-Yong Fan, Ying-Ying Jiang, Xin-Ran Xi, Gan-Yu Nie, Shuang Zhu, Jun-Jie Zhang, Lu Xu

**Affiliations:** School of Pharmacy, Shenyang Pharmaceutical University, Shenyang, 110016, China.

**Keywords:** nanocatalyst, Znproptosis, FDX2/LIAS pathway, Akt/mTOR pathway, cascade amplificated effect

## Abstract

**Background:** Traditional programmed cell death, including ferroptosis, cuproptosis, and apoptosis, has demonstrated excellent anti-tumor effects and declared their complete mechanisms, however, the zinc ion-mediated tumor inhibiting mechanisms remain insufficiently explored. In this study, a self-generated oxygen nanocatalytic system (ZnO@COF@EM, ZCE) was developed to stimulate cascade amplified effect (CAE) of reactive oxygen species (ROS) generation leading to Znproptosis. The underlying Znproptosis mechanism to disrupt mitochondrial (Mito) metabolism was also investigated.

**Methods:** Specifically, the principle of Znproptosis caused by accumulated zinc and ROS, which served as key factors, was declared through western blot analysis and genetic testing. The mechanism of generated ROS (·OH and^ 1^O_2_) under NIR irradiation by ZCE was detected by UV scanning curves, confocal laser scanning microscopy (CLSM) images, and density functional analysis. The injury condition of Fe-S protein of mitochondria metabolism, which triggered Znproptosis with FDX2/LIAS pathway by zinc and ROS, was examined by PCR test and MTT assay. Notably, a Mito-targeting strategy for ZCE was proposed by using molecular docking technology, wherein Zn^2+^ was recognized by zinc finger proteins (ZFPs) with the Mito.

**Results:** ZCE, along with CAE, produced abundant ROS (2.42-time more than control group). At the quantum chemical level, the CAE mechanism was associated with a narrower highest occupied molecular orbital-lowest unoccupied molecular orbital (HOMO-LUMO) gap and increased electronic energy motion within ZCE, which prolonged the excited triplet state (ETS). At the gene level, Znproptosis was achieved by regulating FDX2 and ZIP7 proteins to damage Fe-S protein. The cell cycle was interrupted by Chk2/Cdc25C/Cdc2 and Chk2/p21/Cyclin B1 pathways, leading to the arrest of G1/S and G2/M phases of the cell cycle and inhibition of the Akt/mTOR signaling pathway. Moreover, Znproptosis induced by overloading zinc ions and ROS resulted in a significant antitumor effect (up to 83.81%).

**Conclusion:** Hence, the research reveals a detailed Znproptosis mechanism in nanocatalytic system. Through regulating FDX2/LIAS pathway, Znproptosis could improve the death rate of mitochondria by decreasing the production of Fe-S protein, contributing to advancements in the field of antitumor therapy.

## Introduction

Nowadays, nanocatalysts have been developed to target and kill tumors through Ferroptosis, Cuproptosis and apoptosis to induce cancer cell death 1-3. Fe_3_O_4_, as a self-supplying ROS-responsive system, enhances the therapeutic efficacy of Ferroptosis and immunotherapy by ROS-mediated size reduction and charge reversion 4. PEG@Cu_2_O-ES nano-composites have displayed a strong anti-tumor effect by inducing Cuproptosis and can also reprogram the tumor microenvironment (TME) 5. However, the targeting capacity of unmodified metal-based nanoparticles remains limited and is often accompanied by severe toxicity 6. Compared to these nanoparticles 7-10, ZnO serves as a nanocatalyst with unique capabilities, particularly its ability to target mitochondria (Mito) by interacting with zinc finger proteins (ZFPs) 11-13. In comparison to other Zn-based nanoparticles that tend to accumulate in organs like the liver and kidneys, potentially causing chronic inflammation and placing a substantial burden on the body, ZnO exhibits superior biodegradability and biocompatibility 14. The reported antitumor mechanism of action for Zn-based and other nanocatalytic systems primarily focus on reactive oxygen species (ROS) induction 15, apoptosis 16 and pyroptosis 17, but zinc as a catalytic metal may induce a “Znproptosis” like Cuproptosis due to similar property with copper 18. Therefore, a novel cell death pathway “Znproptosis”, which depend on zinc and ROS to damage the electron transport chain (ETC) of mitochondria is proposed.

Recently, covalent organic framework (COF) identified as a conjugated ROS provider, offer superior electron transfer properties compare to zeolitic imidazolate framework (ZIF) 19-21, making COF an excellent enhancer of nanocatalystic through its ability to maintain an excited triplet state (ETS) and continuously produce ROS 22. To maximize the antitumor effects of nanocarriers, cascade amplification effects (CAE) are widely applied to demolish the resilient core of tumor tissue 23, 24. ZnO, acting as a nanocatalyst, initiates CAE process at the quantum chemical level 25-27. The monomer of COF serves as an ideal ligand to conduct molecular docking. Moreover, COF exhibits high biocompatibility when injected *in vivo* 28. Unfortunately, most nanoparticles are constrained by their reliance on oxygen suppliers (e.g., perfluorohexane), resulting in limited reactive oxygen species (ROS) generation and inconsistent cytotoxic effects 29-31. Moreover, the existence of sequential barriers in tumor sites severely limits penetration depth and circulation time, thereby exacerbating therapeutic challenges 32, 33.

Inspired by predominant morphologies observed during biotic evolution, various decorated nanoparticles (NPs), including PEGylated 34, ligand-connected 35, liposome-coated 36, 37, shape-altered 38, surface-modified 39, and film-coated 40, 41 types, have been developed to promote internalization in tumor sites. Unfortunately, the synthesizing process of specially shaped NPs remains complex 20. The investigations have revealed that erythrocyte membranes can serve as a “camouflage”, effectively facilitating NP penetration through tumor barriers 42, 43. Erythrocyte membrane (EM) as immune-free homologous structure under physiological conditions, protect peptides from immune clearance 44. Additionally, EM modification promotes faster movement of NPs within cells, overcoming intracellular barriers 45. COF-EM films could improve the oxygen production 46. These findings suggests that ZnO NPs decorated with dual-film (COF@EM) have a potential to act as “invisible” ROS bomb with CAE. After escaping lysosomes, these nanoparticles could permeate the intracellular environment, releasing DOX and ROS then killing cancer cells.

**Scheme 1:**
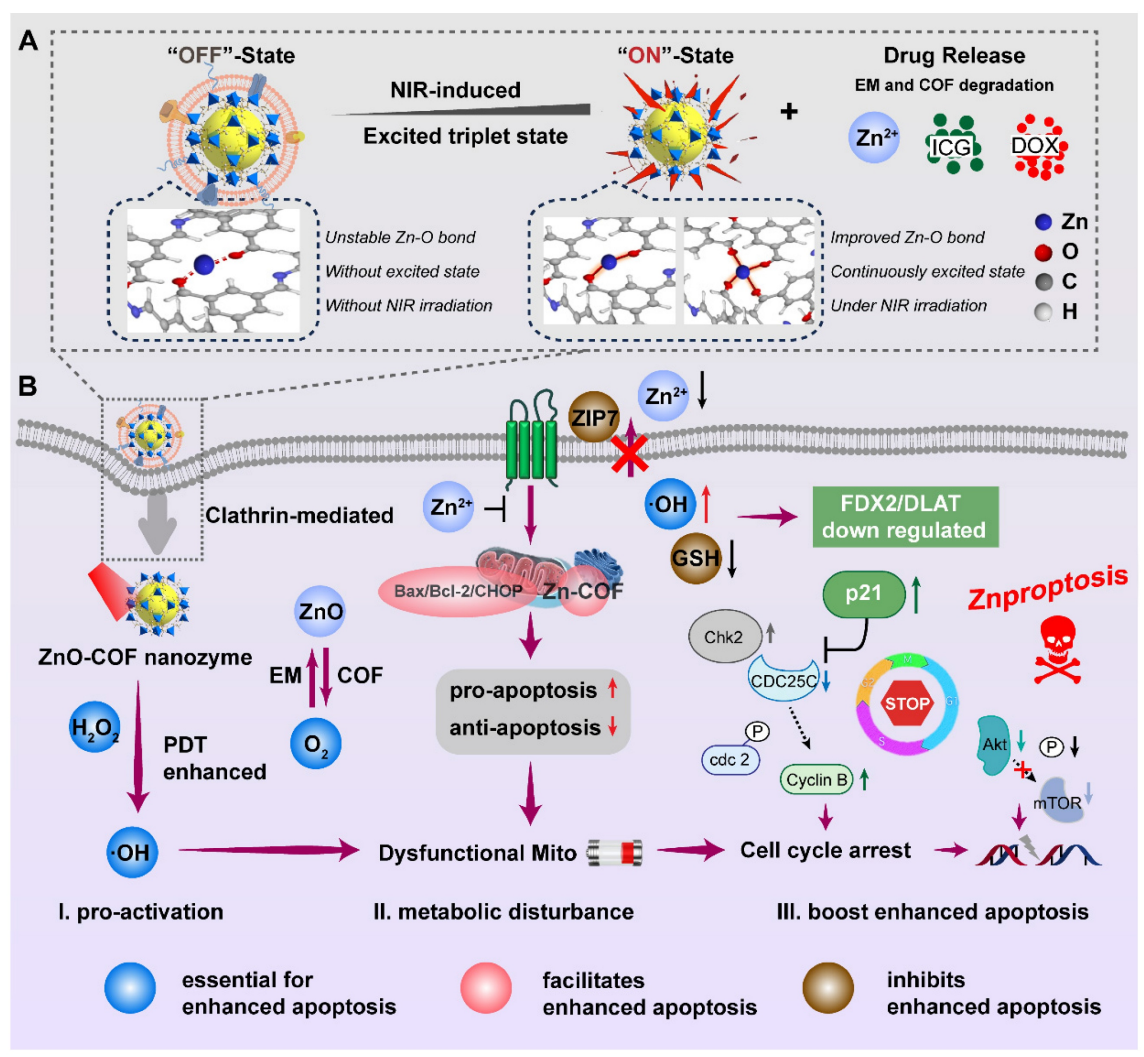
** Schematic nanocatalytic system as a ROS generator with CAE for Znproptosis.** (A) Formation and activation of ZDCIE. (B) Through effective endocytosis process, ZCE decomposition for inducing ROS, promoting dysfunctional Mito, arresting cell cycle and inhibiting Akt/mTOR pathway in cancer cell, triggering Znproptosis ultimately.

Herein, a nanocatalytic platform equipped with COF and EM as a dual-film ROS generator was constructed (ZnO(DOX)@COF(ICG)@EM, ZDCIE). At the same time, the electron motion involved in ROS production, mechanism of Mito transport, and the process of triggering Znproptosis were comprehensively demonstrated. ZnO released Zn^2+^, which catalyzed H_2_O_2_ into ROS such as ·OH, ^1^O_2_ and O_2_^-^ sustainably. COF, linked with Zn^2+^, triggered the ROS production circling reaction by decreasing the gap between highest occupied molecular orbital and lower unoccupied molecular orbital (HOMO-LUMO). As shown in scheme 1, the “OFF” and “ON” states represented the coordination bond dynamics between zinc and COF, where the bond was unstable without NIR irradiation (“OFF”) but became stable under NIR irradiation due to the sustained excited state of COF (“ON”). In the “OFF” state, Zn-O bond between zinc and monomer of COF was unstable while in the “ON” state, Zn-O bond became stable and was significantly increased under NIR irradiation, whether for short (left) or long (right) time periods. The obtained ROS was accumulated at Mito and ER accurately, leading to the high expression of Bax/Bcl-2/CHOP. The interaction between nanocatalysts and Bax/Bcl-2/CHOP proteins was characterized by a large cavity volume (> 55 Å^3^). Moreover, accumulated ROS also induced cell cycle arrest at the G1/S and G2/M phases, further inhibiting the Akt/mTOR pathway for strength apoptosis. In addition, this “Znproptosis” regulated the FDX2/LIAS/ZIP7 signaling pathway to disrupt the Mito metabolism, due to the overload of zinc ions and ROS. EM imparted a bionic function to the nano-system enabling faster permeation through ECM, higher endocytosis rates and longer circulation times. Consequently, the excellent tumor inhibition effect was achieved, and the molecular mechanism was clearly clarified when this nanocatalytic system was applied for tumor treatment.

## Materials and Methods

### Materials

Cobaltous nitrate hexahydrate (Co(NO_3_)_2_·6H_2_O), zinc acetate hexahydrate (Zn(OAC)_2_·6H_2_O), 2-methylimidazole (2-MIM), 1,3,5-Triformylbenzene, 1,4-diaminobenzene, Pimonidazole HCl and 1,3-diphenylisobenzofuran (DPBF) were purchased from Aladdin (Shanghai, China); doxorubicin hydrochloride salt (DOX) was provided by Aladdin (Shanghai, China); DAPI, DCFH-DA, JC-1 kit, lysotracker and mitotracker were purchased from meilunbio (Dalian, China); Bax antibody, Bcl-2 antibody, AnnexinV-FITC/PI apoptosis assays kit was purchased from Wanleibio technology (Shenyang, China); Calcein AM cell viability assay kit, secondary Alexa Fluor 488-conjugated anti-rabbit IgG (H+L), Bortezomib, FDX2 antibody, LIAS antibody, DLAT antibody and Lip-DLAT antibody were purchased from Beyotime Biotechnology (Beijing, China); N-Acetylcysteine (NAC), ML162, Elesclomol, TTM, Fer-1, Nec-1, Rotenone, Antimycin A, FCCP, UK5099, Liproxstatin-1 and KTC1101 were brought from MedChemExpress (Shanghai, China); total RNA was isolated from each sample using the miRNA kit ( Qiagen, Germany); RNA quality was examined by gel electrophoresis and with Qubit (Thermo, Waltham, MA, USA); transmission electron microscopy (TEM) and high resolution transmission electron microscopy (HRTEM) images were obtained on a JSM-6510A transmission electron microscope (JEOL Ltd., Japan); the nitrogen isotherm was obtained on a V-Sorb 2800P surface area and pore size analyzer (Gold App, China); the UV-vis spectra were recorded with a UV-3600 spectrophotometer (Soptop, China); and the fluorescence intensity was measured with a microplate reader (Thermo, USA) ; and confocal laser scanning microscopy (CLSM) images were obtained from confocal laser scanning microscopy (NIS, Germany); electron spin resonance (ESR) spectra were examined on a Bruker ELEXSYS-II E500 CW-EPR; energy dispersive spectrometer (EDS) was performed on a scanning electron microscope (JEM-F200).

### Cell Lines and animals

Mouse breast cancer cell line (4T1) cells were purchased from the Shenyang pharmaceutics university cell bank and were cultured in Roswell Park Memorial Institute (RPMI) 1640 medium with 10% fetal bovine serum and 1% penicillin and streptomycin (100 IU/mL).

Balb/c mice (20 ± 2 g), Balb/c nu mice (20 ± 2 g) and Sprague-Dawley rats (200 ± 20 g) were provided by the Animal Experiments Center of the Liaoning changsheng biotechnology Co. Ltd (operator license number: 21313). All of the animal experiment procedures here were permitted by the Animal Ethics Committee of Sciences.

### Preparation of ZDZIE/ZDCIE as nanocatalytic system

Firstly, ZnO nanoparticles were synthesized* via* solvothermal method according to previous literature with slight modification. Briefly, highly dispersed ZnO nanoparticles were prepared by mixing zinc acetate hexahydrate (1.0975 g) in diethylene glycol (50 mL) at 160 °C for 1 h, which could serve as drug carriers on with the form of mesoporous property. After stirring for 1 h, the NPs were centrifuged for 10 min (10000 rpm) to collect the products. Then, 10 mg of ZnO was dissolved in 3 mL of water and then mixed with 5 mg DOX aqueous solution (3 mL), the mixture was stirred continuously for 12 h. The ZD NPs were gathered by centrifugation (8000 rpm, 10 min) and washing with water three times.

10 mL of ZD NPs methanol solution (5 mg/mL) was mixed with 120 mg cobaltous nitrate hexahydrate. After stirring for 5 min, 2-MIM/methanol solution (10 mg/mL) and 50 μL of triethylamine/methanol solution were dropped. The mixture was continuously stirred for 6 h. The ZDZ NPs were obtained by centrifugation (8000 rpm, 10 min) and washing with methanol three times. Then, 10 mg ZDZ were added into the 5 mL indocyanine green (ICG)/water solution (1 mg/mL) to agitate for 12 h. Finally, the ZDZI NPs were collected after centrifugation (8000 rpm, 10 min) and washing with water three times.

10 mL of ZD NPs acetone solution (1.5 mg/mL) was mixed with 15 mg 1,3,5-triformylbenzene and 8 mg 1,4-diaminobenzene and then 50 μL acetic acid was added. When the solution turned yellow, polyvinylpyrrolidone-40000 (PVP-K30, 0.1 g/mL in acetonitrile) was added, and the mixture was stirred at room temperature for 24 h, the proportion of ZD and COF is 1:1.5. The ZDC NPs were obtained by centrifugation (8000 rpm, 10 min) and washing with acetone three times. Then, 10 mg ZDC were added into the 5 mL ICG/water solution (1 mg/mL) to agitate for 12 h. Finally, the ZDCI NPs were collected after centrifugation (8000 rpm, 10 min) and washing with water three times.

The membranes of erythrocytes were collected from broken erythrocytes, which were separated from whole blood of tumor-bearing mice. EM vesicles were fabricated by sonicating a mixture of NPs and EM (NPs:EM=1:1) in an ice bath for 3 min using ultrasonic cell disruption system with 2W (SCIENTZ-IID, China).

### Characterization of nanocatalytic system

Particle size and zeta potential were tested by a NanoZS (Malvern, UK). The structure of membrane coating ZnO was conducted by confocal laser scanning microscopy (CLSM, NIS-Elements, Japan), transmission electron microscope (TEM, HT7800, Hitachi, Japan) and cryogenic transmission electron microscope (Cyro-TEM, Regulus8100, Hitachi, Japan). To confirm the membrane proteins of EM vesicles, ZZE and ZCE samples with equivalent protein amounts were loaded onto a 10% sodium dodecyl sulfate-polyacrylamide delelectrophoresis (SDS-PAGE) gel (Dalian Meilun Biotech Co. Ltd., China) and run. The collect gel was subsequently imaged using a AI600 gel documentation system (GE, China) after staining in coomassie blue. Plasma protein absorption of NPs was analyzed by a similar procedure after incubation with blood plasma for 4 h. To character the chemical element of NPs, the ^1^HMR FTIR, UV-vis spectroscopy scan and X-ray photoelectron spectroscopy (Thermo Scientific K-Alpha, USA) were applied to describe the composed of NPs. The chemical composition and element valence were analyzed using X-ray photoelectron spectroscopy (XPS, Thermo Scientific K-Alpha, American). The electron spin resonance (ESR) spectra were examined on a Bruker ELEXSYS-II E500 CW-EPR to detect the production **·**OH and ^1^O_2_.

### Evaluation of CAE condition and mechanism by quantum chemical theory

Methylthionine chloride and 1,3-Diphenylisobenzofuran (DPBF) as ROS sensors were used to detect the amount of extracellular ROS. Briefly, 10 μg/mL methylthionine chloride was added to 5 mL NPs/water solution (50 μg/mL or different concentrations) with or without H_2_O_2_ (40 μM). Then, the mixture was kept in the dark or irradiated with an 808 nm laser for 10 min to arrive the excited triplet state (ETS). The absorbance intensity of methylthionine chloride and DPBF at 666/424 nm were measured every 5 min for detecting ROS with CAE. Moreover, ETS of NPs under abundant ROS was measured by UV-vis spectrophotometer (UV-9600, China).

For ESR detection, DMPO served as the spin-trapping agent for hydroxyl radicals (•OH) and singlet oxygen (¹O₂). ZCE (1 mg) was added into the weak acidic buffer (pH 6.5) containing H_2_O_2_ (1.0 mL) and 100 μM DMPO. Then, the resulting mixture were transferred to a quartz tube for ESR assay after mixing by sonication for 1 min.

For ROS with CAE detection at intracellular and tumor, 2′,7′-dichlorofluorescein diacetate (DCFH-DA) was exhibited as a ROS probe. 4T1 cells were seeded in a twelve-well plate with coverslips for 12 h and then incubated with different NPs for another 4 h. After that, DCFH-DA (10 μM) was added into the cells to incubate for 30 min and irradiated with an 808 nm laser (2 W/cm^2^) for 5 min. At last, the cells were observed by CLSM (NIS-Elements, Japan).

For demonstrated the energy transition behavior in molecular orbitals of COF and ZCE which was the mechanism of CAE between Zn and COF, the electronic structures for 5-(((4-aminophenyl)imino)methyl)isophthalaldehyde (monomer of COF-LZU-1) and its Zn complex were studied *via* density functional theory (DFT), where all geometries were optimized by the PBE0 functional and def2-TZVP basis set *via* Gaussian 16 C.02 package. Harmonic vibrational frequency was performed at the same level to guarantee that there is zero imaginary frequency for minima. The highest occupied molecular orbital (HOMO) and lower unoccupied molecular orbital (LUMO) were calculated by Multiwfn 3.8 (dev), whose input files were extracted from Gaussian formatted checkpoint files, and plotted by VMD 1.9.3.

### Self-oxygen generation ability

In order to conduct the hypoxia atmosphere, 2 mL of liquid paraffin was added into the solution of samples (10 mL, 50 μg/mL), the mixture was degassed by vacuum pump and bubbled with nitrogen for 15 min. Then, different amounts of H_2_O_2_ (0, 1, 20, or 40 μM) and NPs (200 μg/mL) were added to the solution and the real-time generation of O_2_ was measured by a DO9-100 dissolved oxygen analyzer.

The intracellular O_2_ was further investigated using hypoxia detection factor including Pimonidazole HCl and HIF-1α. 4T1 cells were seeded in a twelve-well plate under normoxic (21% O_2_) or hypoxic (1% O_2_) conditions for 12 h and then cells/tumor tissues were incubated with NPs (200 μg/mL) for another 4 h. Afterward, the Pimonidazole HCl (10 μM) was added into the cells to incubate for 2 h and then the cells were observed by CLSM. Moreover, for HIF-1α, it was necessary to conduct the immunofluorescence for the cells. The cells were fixed with 4% paraformaldehyde for 20 min and washed with PBS three times. The cells were permeabilized with 0.1% Triton X-100 at 25 °C for 5 min. After washing with PBS, the cells were blocked with PBS containing 3% bovine serum albumin at 25 °C for 2 h. Then, the primary anti-HIF-1α antibody was added and incubated at 4 °C for 12 h. The cells were washed three times with PBS and incubated with the secondary Alexa Fluor488-conjugated goat anti-rabbit IgG H&L antibody for 1 h. The sample was washed thrice with PBS and stained with 4′,6-diamidino-2-phenylindole (DAPI, 10 μM) for 15 min. Finally, the cells were observed by CLSM.

### DFT calculation

Our spin-polarized density functional theory (DFT) calculations were carried out in the Vienna ab initio simulation package (VASP) based on the plane-wave basis sets with the projector augmented-wave method. The exchange-correlation potential was treated by using a generalized gradient approximation (GGA) with the Perdew-Burke-Ernzerhof (PBE) parametrization. The van der Waals correction of Grimme's DFT-D3 model was also adopted. A vacuum region of about 15 Å was applied to avoid the interaction between adjacent images. The energy cutoff was set to be 450 eV. The Brillouin-zone integration was sampled with a Γ-centered Monkhorst-Pack mesh^7^ of 1 × 1 × 3. The structures were fully relaxed until the maximum force on each atom was less than 0.04 eV/Å, and the energy convergent standard was 10^-6^ eV. To accurately calculate the band structure, the number of the inserting points was set to 20.

### *In vitro* drug release

5 mg of NPs were dispersed into 5 mL PBS solutions (pH 6.5 and pH 7.4) and placed in dialysis bag (intercept molecular weight 8000-12000), then shaken at 37 °C (100 rpm) for 72 h. At the period time, 2 mL of release media were collected by centrifugation and replaced with the same volume of fresh media in the 20 mL media system. The release of DOX and ICG from the ZDCIE nanoplatform was measured by a UV-vis spectrophotometer.

### Multiple particle tracking (MPT)

MPT experiments were conducted as previously described, and collagen I was regarded as the simulated ECM structure. The pattern of carboxymethylcellulose has a fibrosis network and similar pore size compared to ECM, as revealed by scanning electron microscopy in the literature. Briefly, the motion curve of the particles was observed using a fluorescence microscope (AX, Nikon, Japan) with a 40-fold microscope and recorded the video for 10 s. The collected data were processed using Image J software. The time-mean square displacement (MSD) and effective diffusivity (D_eff_) were calculated by the following equations (1) and (2):


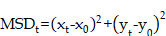
 (1)


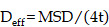
 (2)

where x and y are the coordinates of the particles, and t is the time.

### Cellular uptake and toxicity

For cellular uptake, 4T1 cells were seeded in a twelve-well plate with coverslips for 12 h and then incubated with different FITC labeled NPs (ZD, ZDZ, ZDC, ZDZE, ZDCE, 200 μg/mL) for another 8 h. Afterwards, the cells were fixed with 4% paraformaldehyde for 20 min and washed with PBS three times. Then, the samples were stained with DAPI for 15 min, washed with PBS three times and observed by CLSM. Additionally, the cells were collected for quantitative analysis using BCA method and flow cytometry (BD, America).

For cellular toxicity, 4T1, Hela and HepG-2 cells were seeded in a 96-well plate with coverslips for 12 h and then incubated with different NPs (ZD, ZDZ, ZDC, ZDZE, ZDCE) for another 48 h at 37 °C. The DOX concentrations were 0, 2, 10, 20, 40 and 50 μg/mL. After that, the cells were irradiated with an 808 nm laser (2 W/cm^2^) for 5 min or in the dark. The MTT assay was performed to evaluate the cytotoxicity of DOX-loaded NPs, free DOX and blank NPs to 4T1 cells followed the standard protocol.

### Permeation in multicellular tumor spheroids (MTSs)

MTSs with uniformed size composed of 4T1 cells were obtained and incubated with RITC labeled NPs (ZDZIE and ZDCIE) for 4 h. The MTSs were placed to confocal dishes to observe the distribution of NPs, and the penetration depth of the MTSs was observed by CLSM.

### *In vitro* antitumor efficacy

The capacity of NPs to antitumor *in vitro* was investigated using the Live-Dead staining assay. 4T1 cells were seeded in a 12-well plate with coverslips, and multicellular tumor spheroids were cultivated for 12 h and then incubated with NPs for another 4 h. After that, the 4T1 cells were irradiated with an 808 nm laser (2 W/cm^2^) for 5 min or incubated in the dark. Then, the samples were incubated with calcein acetoxymethyl (AM) for 20 min and PI for 5 min. Finally, the cells were observed by CLSM.

### *In vivo* circulation time study

The determination of *in vivo* circulation time of NPs was investigated using SD rats as model animal as previous method. The FITC labeled NPs (2 mg/kg) were injected intravenously from tail vein. The 150 μL blood samples were collected form preparative time and then centrifuged at 4000 rpm to separate the plasma for further determination. The FITC fluorescence intensity was then measured to determine the blood circulation of NPs.

### Organization distribution of NPs

Tumor model was conducted by injecting 4T1 cells into the subcutaneous tissue of Balb/c mice. ICG-labeled NPs (30 μg/mL) were injected via the tail vein. Fluorescent images were obtained at time points with an IVIS 100 Spectrum system (PerkinElmer, USA), and images were analyzed by Living Image software.

### Intracellular trafficking and targeting capacity study by molecular docking technology

To describe the motion state of NPs intracellular, FITC labeled NPs (ZDZIE and ZDCIE) were co-incubated with 4T1 cells and observed by CLSM. The lysosome and mitochondrion were labeled with LAMP1 rabbit mAb and Mito-Tracker Green. The cells were fixed with 4% paraformaldehyde for 20 min and washed with PBS three times. The cells were permeabilized with 0.1% Triton X-100 at 25 °C for 5 min. After washing with PBS, the cells were blocked with PBS containing 3% bovine serum albumin at 25 °C for 2 h. Then, the primary antibody was added and incubated at 4 °C for 12 h. The cells were washed three times with PBS and incubated with the secondary Alexa Fluor488-conjugated goat anti-rabbit IgG H&L antibody for 1 h. The sample was washed thrice with PBS and stained with 4′,6-diamidino-2-phenylindole (DAPI) for 15 min.

### Mitochondrial dysfunction condition measurement

The JC-1 kit was applied to measure the potential of mitochondrial membrane. 4T1 cells were seeded in a 12-well plate with coverslips for 12 h and then incubated with NPs (control, ZDZIE and ZDCIE) for another 4 h. Then the cells were irradiated with an 808 nm laser (2 W/cm^2^) for 5 min or in the dark. After staining with JC-1 (2.5 μg/mL) for 30 min at 37 °C, the samples were analyzed by CLSM.

### ZCE-Bax/Bcl-2/CHOP apoptosome detection

To evaluate ROS induced ER/Mito stress, the expression of Bax/Bcl-2/CHOP was measured. 4T1 cells were seeded in a 12-well plate with coverslips for 12 h and incubated with NPs (control, ZDZIE and ZDCIE) for another 4 h. Then the cells were irradiated with an 808 nm laser (2 W/cm^2^) for 5 min or in the dark and incubated with Bax/Bcl-2/CHOP-specific antibody as procedure statement before 47. Finally, the samples were analyzed by CLSM. The formation progress of ZCE- Bax/Bcl-2/CHOP apoptosome and their linkage pattern were detected by molecular docking technology with Discovery Studio software (2019).

### Bio-TEM

The 4T1 cells were incubated with ZCE for 24 h. After that, cells were washed with PBS for 3 times and further fixed with 2.5% glutaraldehyde in 0.03 M potassium phosphate buffer for more than 24 h. Finally, the images were obtained on JEOL2100 electron microscope.

### Znproptosis mechanism detection

For RNA-sequencing, 1 μg of total RNA was used to prepare small RNA library. Sequencing libraries were generated using VAHTSTM Total RNA-seq (H/M/R) Library Prep Kit for Illumina®. The libraries were sequenced as 51-bp paired-end reads using Illumina Novaseq6000 according to the manufacturer's instructions by the commercial service of Genergy Biotechnology Co. Ltd. (Shanghai, China). The raw data of all samples had been submitted to the Sequence Read Archive at the National Center for Biotechnology Information.

The clean data were aligned to the miRBase database using Bowtie version 1.0 to obtain the miRNAs expression counts. The CPM values were calculated by edgeR version 3.22.5.Differentially expression analysis between matched tumor and normal samples were performed by the R package DESeq version 1.32.0. Differentially expressed genes exhibiting two-fold changes and P values ≤ 0.05 were selected.

The evaluated process for expressions of ZIP7 and FDX2 with HIF-1α by using anti-body labeled and observed by CLSM.

### Immune system regulated experiments and expression of relevant immune factors

For immunological evaluation *in vivo*, tumor models were established as described above. When the tumor size reached approximately 100 mm^3^, tumor-bearing mice (n = 5) were intravenously administrated saline and NPs with dosage of 2 mg/kg. Afterwards, mice were sacrificed on day 7 and centrifuged at 3000 rpm 15 min. The supernatant was collected for IL-2, IL-4, IL-6, IL-10, IL-12, IL-15, IL-18, IL-1β, TGF-β, IFN-γ and TNF-α analysis via corresponding assay kit mentioned above. The pellet was added with red blood cell lysis buffer, and washed with PBS once to obtain single-cell suspensions. To stain the CD8^+^, CD45+ T cells, DC cells, MSDC cells and M1/M2 cells, single-cell suspensions were first incubated with 2.5 μg/mL anti-CD16/32 antibody in 5% BSA solution at 4 ◦C for 60 min to block the nonspecific Fc interaction, and then incubated with 5% BSA solution containing fluorescent-labeled primary antibodies of 2.5 μg/mL anti-CD107a-PE/CD8-APC, anti-CD45-APC, anti-Gr-1-PE/CD11b-APC, anti-CD86-APC and anti-CD86-PE/CD206-APC at 4 ◦C for another 60 min. Afterwards, cells were washed and suspended in PBS for flow cytometry analysis.

### *In vivo* antitumor efficacy

The 4T1/Hela/HepG2 tumor-bearing Balb/c mice (ectopic and *in situ*)/Balb/c nu mice were as animal model to evaluate the antitumor efficacy of NPs. When the value of tumor volume reached about 100 mm^3^, the mice were randomly divided into the following 9 groups (n = 5): (1) control, (2) free DOX, (3) ZD, (4) ZDCI, (5) ZDCI+laser, (6) ZDCIE, (7) ZDCIE+laser, (8) control (*in situ*), and (9) ZDCIE+laser (*in situ*). Every tumor-bearing mouse received DOX-loaded NPs injection (2 mg/kg) every other day. After 12 h of intravenous injection, the mice in groups 5, 7, 9 were irradiated with an 808 nm laser for 10 min (2 W/cm^2^). Tumor volume and body weight of mice were recorded every other day. After 14 days of treatment, the mice were sacrificed and the tumors and major organs were collected for further analysis.

For TUNEL and Ki-67 staining, 4T1 cells were smeared after fixation, blocked and permeabilization and then were incubated with TUNEL reaction mixture (TUNEL Apoptosis Assay Kit) in the dark at 37 °C for 1 h. After which, cells were washed with PBS for three times and then imaged by a florescent microscope (Evos FL Colour Imaging System from Thermo Scientific). After 14 days' treatment, tumors and main organs were collected from the sacrificed mice for TUNEL and Ki67 staining.

For histological examination(H&E) staining, the tissues of liver, lung, kidney, heart, spleen and tumor of NPs treated mice were excised and sliced into less than 1 cm × 1 cm, dehydrated successively with buffered formalin, ethanol of different concentrations, and xylene in turn after 14 days of treatment. Finally, all dehydrated tissues were embedded in liquid paraffin, sections were stained with hematoxylin and eosin (H&E), and observed using an optical microscope.

### Biocompatible analysis

Kunming mice injected with the PBS and ZCE were sacrificed at 7 and 90 days for blood collection. Then, the collected blood samples were further treated with anticoagulants and followed by centrifugation to yield the blood plasma for analysis. The serum biochemistry data including aspartate transaminase (AST), alanine transaminase (ALT), alkaline phosphatase (ALP), total protein (TP), blood urea nitrogen (BUN), creatinine (CRE), white blood cells (WBC) and so on were analyzed by diagnostic kits.

### Statistical analysis

Statistical significance was analyzed by a two-sample Student's t test and one-way ANOVA when comparing multiple groups, which shown as ****p < 0.0001, ***p < 0.001, **p < 0.01, and *p < 0.05.

## Results and Discussion

### Synthesis and characterization of nanocatalytic system

In this study, we developed multifunctional nanocatalysts with varying frameworks and compositions. The core-shell structure was composed of ZnO-ZIF/COF-EM. Firstly, the spherical ZnO core was fabricated by wet chemical method. The first layer shell, a framework layer, acted as a “nanofence” to assist the core in generating ROS (designated as ZZ and ZC). Then, the second shell, an EM, was obtained from the tumor-bearing Balb/C blood by supersonic lysing method combined with ultracentrifugation. Finally, the NPs called ZZ and ZC were coated with EM followed by ultrasonic method forming ZZE and ZCE.

**Figure 1:**
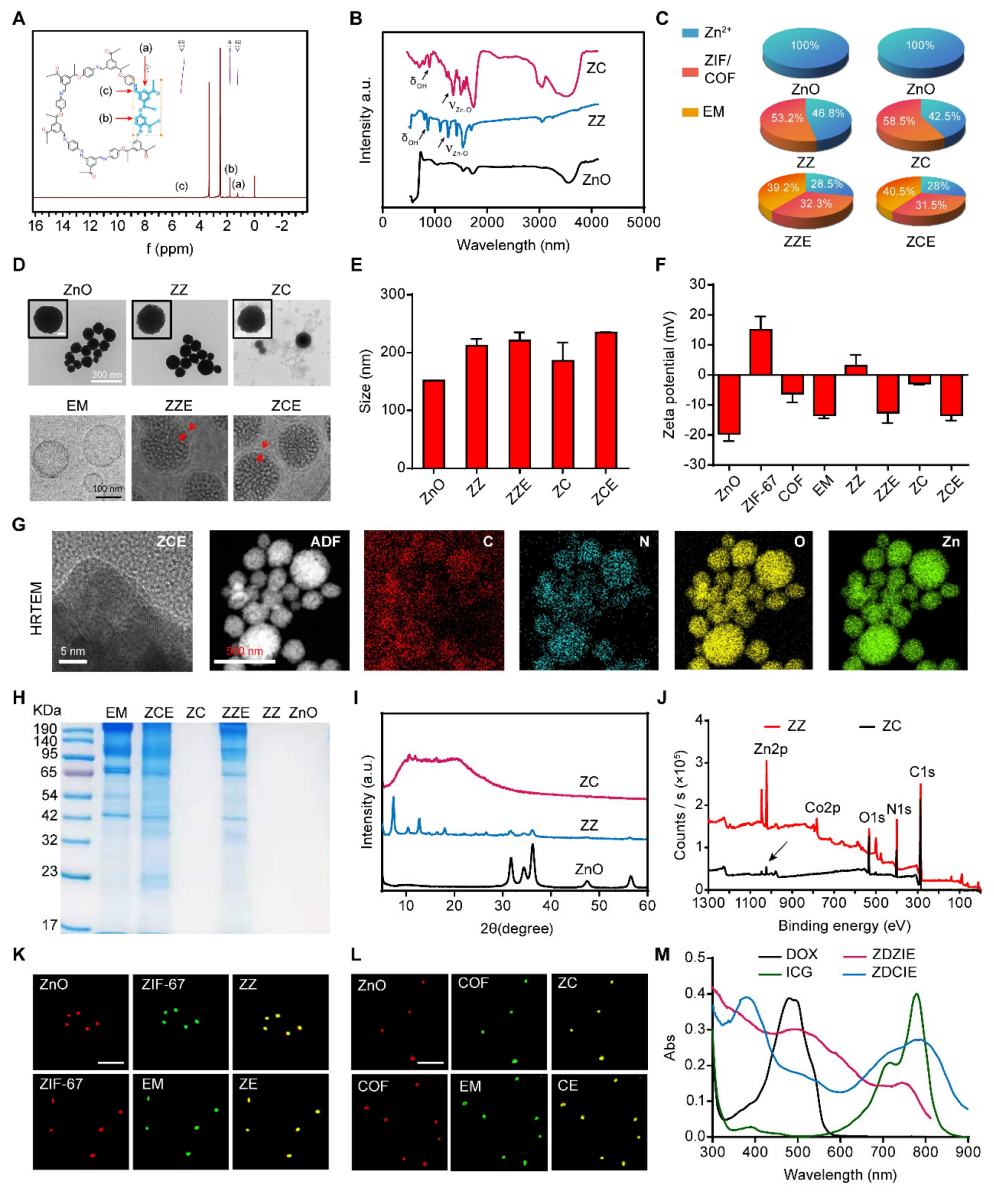
** Characterization of NPs.** (A) ^1^H NMR spectra of COF; (B) FTIR spectra of ZnO, ZZ and ZC; (C) Different composed fractions distribution of ZZE and ZCE; (D) TEM images of ZnO, ZZ and ZC. Scale bar is 300 nm; and Cyro-TEM images of EM, ZZE and ZCE (The red rows are labeled the EM films). Scale bar is 100 nm. (E) The sizes and (F) zeta potentials of NPs; (G) The HRTEM images of ZCE and elemental mapping images of C, N, O and Zn. Scale bar, 5 nm and 500 nm. (H) SDS-PAGE analysis of EM, ZCE, ZC, ZZE, ZZ and ZnO; (I) XRD patterns of ZnO, ZZ and ZC; (J) XPS patterns of ZZ and ZC; (K) The CLSM images of ZZ, ZE and (L) ZC, CE, red, RITC-labeled; green, FITC-labeled. Scale bar, 1 μm; (M) UV-vis spectra of DOX, ICG, and prepared NPs.

COF, a novel conjugated polymer with a more excellent potent tumor inhibition effect than ZIF-67, was selected as a photosensitizer enhancer to form a tight shell around the ZnO core, while ZIF-67 was selected as a control. As shown in Figure 1A, the exact structure of the synthesized COF-LZU-1 confirmed using ^1^H NMR spectroscopy, where characteristic peaks of COF-LZU-1 were identified 20, 48. Fourier transform infrared spectroscopy (FTIR) was conducted to further investigate the linkage between Zn and COF/ZIF-67. The symmetry and spatial geometry of ZnO were altered after coordination with COF or ZIF, leading to the formation of new coordination bonds. The stretching and bending vibrations of coordinated Zn-O bonds were observed at ~1450 cm^-1^ and ~ 1683 cm^-1^ for ZZ and ZC, confirming the successful preparation of these compounds. Additionally, the peaks at ~967 cm^-1^ and ~850 cm^-1^ corresponding to δ(O-H), further supported the existence of coordination bonds between Zn and ZIF or COF (Figure 1B and Table 1). The detailed amounts of ZnO present as Zn ions in ZCE and ZZE were depicted in Figure 1C.

The size and morphology of NPs were exhibited as Figures 1D and 1E. According to the images of TEM the samples appeared as nanosphere with an approximate diameter of 150 nm. The size of uncoated ZnO NPs was about 151 nm, while the dual-film coating of ZIF-67/COF and EM, resulted in slightly increased in the diameters of ZZ, ZZE, ZC and ZCE to 212 nm, 220 nm, 186 nm and 236 nm, respectively. Upon coating ZnO with the first film of ZIF-67 or COF, the zeta potentials shifted from -20.6 mV to 4.8 mV and -2.8 mV, respectively, showing slight differences compared to ZIF-67 (10.8 mV) and COF (-6.1 mV). After the addition of the second EM film, the zeta potentials of ZZE and ZCE became similar to that of EM, indicating successful decoration of the NPs with the EM layer (Figure 1F). The existences of Zn, O, N, and C elements in HRTEM-EDS mapping images and EDS curve verified the successful preparation of ZCE (Figures 1G and S1). In order to ensure the integrity of proteins on the surface of ZZE and ZCE, the sodium dodecyl sulfate polyacrylamide gel electrophoresis (SDS-PAGE) analysis was conducted. The protein profiles of ZZE and ZCE closely matched those of erythrocyte membrane (EM), and the major protein components from the EM were preserved in both ZZE and ZCE (Figure 1H). In brief, the dual-film coated NPs were fabricated completely. The crystal structures of ZnO core and NPs were further confirmed by X-ray diffraction (XRD) analysis. As shown in Figure 1I, all diffraction peaks of ZZ and ZC indicated a similar single crystal structure originating from ZIF-67 or COF, revealing that the crystallinity was altered by the shell modification. Moreover, XPS spectroscopy also verified the stable first-film coating of ZZ and ZC NPs with the characteristic peaks for Zn, Co, C, N and O (Figure 1J). The overlapping fluorescent signals further verified the integrity of ZZE and ZCE, confirming the core-shell structure of the NPs (Figures 1K and 1L). The BET curves of ZnO, ZZ and ZC reflected that the existence of small pore including 7.7 nm, 5.5 nm and 4.3 nm, respectively, which were capable of loading molecules (Figure S2). UV-vis absorbance and emission spectra clearly exhibited the characteristic peaks of DOX and ICG, and after loading drugs, the sizes and zeta potentials of nanoplatform were not changed significantly, demonstrating that the drug was successfully loaded into the pores of NPs (Figures 1M and S3).

### Catalytic activity and ROS production with CAE mechanism

**Figure 2:**
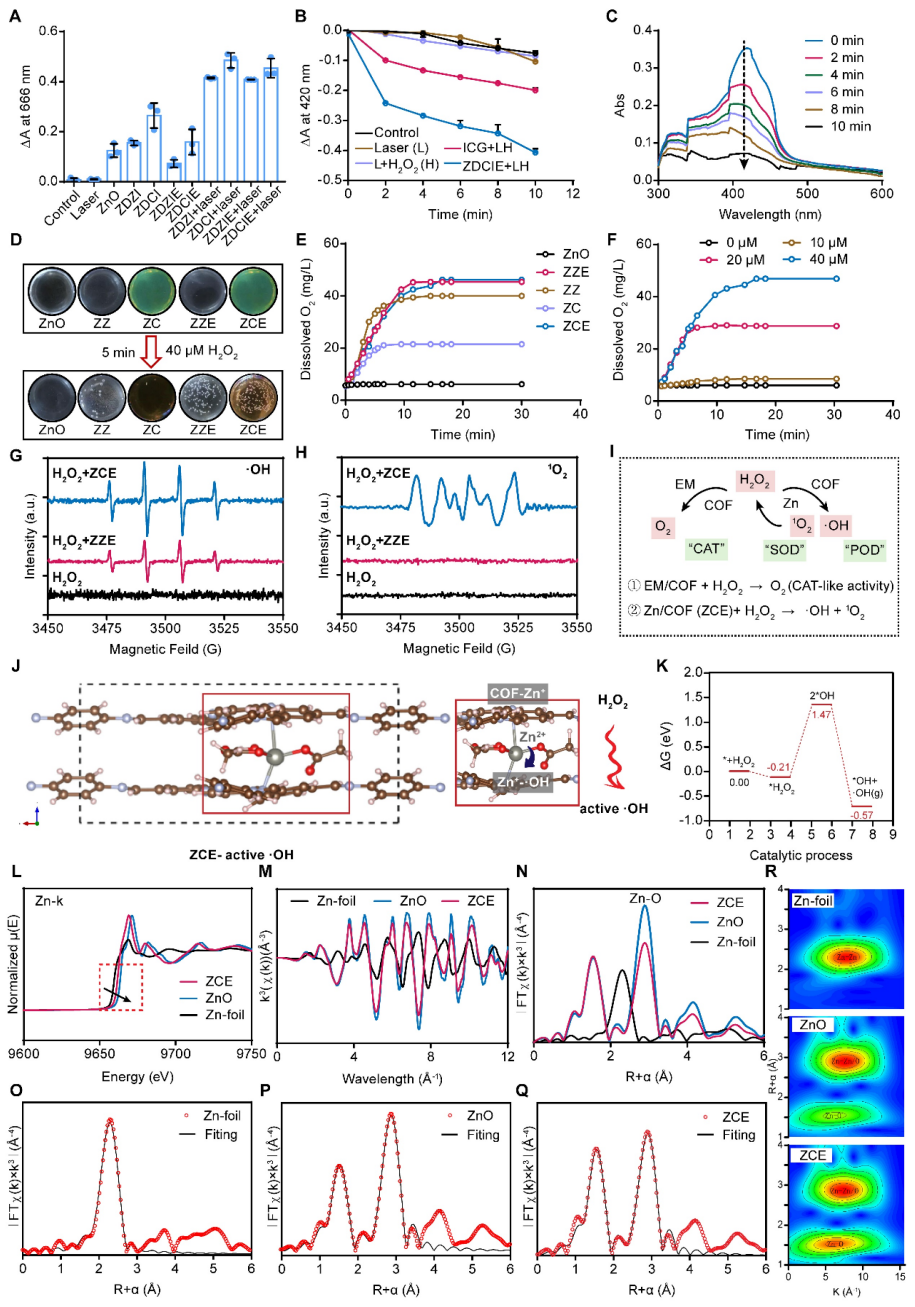
***In vitro* self-oxygen generating capacity and ROS generation mechanism studies.** (A) Absorbance changes of methylene blue incubated with NPs with or without laser irradiation for 10 min; (B) Absorbance changes of DPBF incubated with ZDCIE with or without l/H (laser/H_2_O_2_ (40 μM)); (C) UV-vis spectroscopy of DPBF incubated with ZDCIE with laser irradiation for 0, 2, 4, 6, 8 and 10 min; (D) O_2_ generation images of NPs; (E) O_2_ concentration changes of NPs; (F) O_2_ concentration changes of ZZE solutions after incubated with various concentrations of H_2_O_2_; ESR spectra for detection of ·OH (G)and ^1^O_2_ (H) in different conditions by DMPO. (I) Schematic illumination of mechanism for ZCE to produce O_2_ and ROS; (J) The produced progress of active ·OH by the interaction between COF and Zn. (K) Energy changing curves during the catalytic process. (L) Normalized EXAFS of Zn K-edge spectra (E-space); (M) k^3^χ(k) space spectra fitting curve of Zn foil, ZnO, and ZCE (k-space), and (N) Fourier transforms of k^3^-weighted Zn K-edge EXAFS spectra (R-space); FT-EXAFS fitting curves at R space of Zn foil (O), ZnO (P) and ZCE (Q); (R) WT-EXAFS plots of Zn-foil, ZnO and ZCE. The data are presented as means ± SD, n = 3, *p < 0.05, **p < 0.01 and ***p < 0.001.

To investigate ROS generation ability of ZDZI, ZDZIE, ZDCI and ZDCIE, the methylene blue (MB) was displayed as an indicator to detect the amounts of **·**OH by recording the decrease in absorbance at 666 nm (Figure S4). As shown in Figure 2A, the absorbance intensity of MB at 666 nm significantly decreased after treatment with dual-film ZnO NPs (ZDCIE) with 808 nm laser irradiation for 5 min. On the contrary, less absorbance intensity changes were observed in the control group, laser group and samples without irradiation. As the concentrations of ZDZIE and ZDCIE increased, the absorbance intensity dropped by approximately 17.7% and 31.7% respectively. This decreased was slightly lower compared to ZDZI and ZDCI, likely due to the EM coating, which promotes internalization (Figure S5). The above results showed that ZDCIE could produce abundant **·**OH with the energy transitions (type one reaction) of COF film, while ZDZIE also displayed significant **·**OH generation, although slightly less than ZDCIE. Furthermore, for verifying ROS generation ability of NPs, the DPBF, whose absorbance intensity at 420 nm, decreased irreversibly after reacting with ROS, was employed as a ^1^O_2_ sensor. Similar to the MB results (Figure 2B), when H_2_O_2_ was added to stimulate the tumor microenvironment, a corresponding decrease in the UV absorption peak of DPBF was observed with increasing irradiation time under the 808 nm laser. The peak gradually disappeared incubated with ZDCIE, suggesting a great number of ROS were released (Figure 2C). Afterward, the CAE between ROS and ZC with excited triplet state was activated. As shown in Figure S6, compared to free ZnO, after 8 h, ZC (ZnO@COF) exhibited a wider and more stable band suggesting that the excited triplet state was prolonged, largely due to the CAE. It is well known that the main kind of ROS is ^1^O_2_, which originates from type two reaction between oxygen and photosensitizer 49. To investigate oxygen generation capacity, a dissolved oxygen meter was used after mixing the NPs and incubating with 40 μM H_2_O_2_. In Figure 2D, the dissolved oxygen levels of dual-film NPs ZZE and ZCE increased rapidly in the simulated tumor environment (40 μM H_2_O_2_), the dissolved O_2_ were 42 mg/L (Figure 2E). Especially, as the concentration of H_2_O_2_ increased, oxygen levels rose increased slowly when the substrate was insufficient, but increased rapidly inadequate substrate environments (Figure 2F). Then, ESR spectra was adopted to verify the formation of **·**OH and ^1^O_2_ of ZCE in different conditions using 5,5-dimethyl-1-pyrroline N-oxide (DMPO) as the spin-trapping agent. The straight line was detected in H_2_O_2_ group, indicating the absence of **·**OH generation. In ZCE group with NIR, the ESR spectra had several lines with an intensity ratio of 1:2:2:1, indicating the generation of **·**OH caused by the active ZCE during the Fenton-like reaction (Figure 2G). In Figure 2H, mMn/SAE displayed a strong signal with an intensity ratio of 1:1:1:1, indicating ZCE as a POD-like nanozyme could catalyze O_2_ into ^1^O_2_.

It was obvious that the existence of H_2_O_2_ in tumor could trigger the catalyzers (COF) to self-generate O_2_, further providing the necessary materials for ROS production by ZnO and ICG under laser irradiation (Figure 2I). The reaction rate curves were constructed based on the kinetic theory that the curves for O_2_ and ROS curves followed an “S” shaped pattern, characteristic of a catalytic reaction, resembling the curve of a consecutive reaction. The yields of O_2_ and ROS gradually increased, primarily generated by ZCE under NIR (Figure S7A). The catalytic mechanism of ZCE, functioning as a nanocatalytic system, could be explained using typical catalytic reaction rate equations (3) and (4) 50:


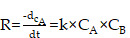
 (3)


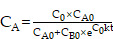
 (4)

where R is reaction rate; k is a constant; C_A_, C_B_ is the concentration of A and B; C_0_ is the initial centration of medium; C_A0_, C_B0_ is the initial concentration of A and B; t is time.

Additionally, EM-film could assist COF to provide O_2_, attributed to the existence of myoglobin in EM. Taken together, this sufficient O_2_ nanoplatform show great potential for promoting ROS accumulation through the CAE in ZZIE and ZCIE, involving both type one & two reaction 51. In the type one reaction, the excited ZCIE transferred electrons to atoms, raising them to higher energy levels and catalyzing the reduction of O_2_ to release **·**OH, O_2_^-^, and so on. The type two reaction directly generated ^1^O_2_ with the assistance of H_2_O_2_ and O_2_. As shown in Figure S7B, under NIR irradiation (808 nm), ROS were produced continuously, inducing the excited triplet state (ETS) of ZCE, which exhibited a broadened absorption wavelength across three cycles. The three cyclical curves confirmed that the existence of CAE between ETS and ROS. The mechanism by which ZCE produced more ROS, inspired by CAE, involved the broadening of the absorption band to maintain the excited triplet state for an extended period. In order to describe the changes in electronic energy during ROS production, density functional theory (DFT) calculations were performed at the def2-TZVP level. Based on the quantum chemistry theory, the HOMO-LUMO gaps for COF and ZCE (CAE) were calculated to be 3.502 eV and 3.294 eV (Figure S7C), respectively. This reduction in the HOMO-LUMO gap, indicated that CAE promoted increased ROS production by COF. In detailed, the excited photosensitizer could theoretically generate hydroxyl radicals (**·**OH) by electron transfer (type one, S_1_→T_1_) and singlet oxygen (^1^O_2_) by the energy transfer (type two, T_1_→S_0_), aligning with HOMO-LUMO energy levels 52, 53. Using equations based on electron conservation, the atom and electron transfer mechanisms were further explored through computational chemistry. The interactional hypothesis proposed in Figure S7D was as follows: the ZCE1 of ETS reacts with O_2_ to generate ^1^O_2_ and ZCE2, and then the ground state ZCE1 reacts with ^1^O_2_ to produce ZCE2 and **·**OH (Reactions ① and ②), forming a cycle driven by CAE 54. Taken together, the catalytic mechanism of ZCE NPs could be explained as follows: firstly, a Fenton-like reaction occurred, H_2_O_2_ adsorbed the accepts electrons from surface Zn^2+^ and COF, dissociating into active **·**OH. Secondly, the adjacent Zn and COF transferred its electrons on the surface of atoms to form Zn^+^-**·**OH-COF chain, providing the continuous dynamic for catalytic reaction for CAE (Figure 2J). The relative adsorption energy to form active **·**OH was calculated to be -0.57 eV (Figure 2K).

To confirm the coordination environment of Zn on nanocarriers at an atomic level, extended X-ray absorption fine structure (EXAFS) spectroscopy at the Zn K-edge were investigated, Zn foil was displayed as control group. The EXAFS spectra showed that the absorption edge of Zn K-edge lay between that of Zn foil and ZnO, indicating that the valence state of Zn in ZCE was between Zn foil and ZnO (Figure 2L). As shown in Figure 2M, the oscillations of ZCE curve are smooth and basically the same as ZnO, indicating that the coordination structure was same with ZnO. The formation of Zn-O bonds was verified by the fourier transformed (FT) k^3^-weighted EXAFS. The major peak at 2.3 Å belonged to Zn-Zn coordination in Zn-foil group. The peaks in ZnO and ZCE groups at 2.88 Å belonged to Zn-O coordination. Additionally, the peak locations of the ZCE and ZnO were same, indicating that the coordination configuration was same as ZnO (Figure 2N). As shown in Figure 2O, the curve-fitting results matched well with the experimental data and fitting results suggested that ZCE were mainly composed of Zn-O and Zn-Zn coordination. The EXAFS fitting parameters and the Zn-O average coordination numbers for ZCE were calculated to be 4 (Table S2). Moreover, WT-EXAFS analysis of Zn foil, ZnO, and ZCE was performed to verify the coordination condition of ZCE (Figure 2R). Notably, the WT contour plots of ZCE had two intensities at 6.5 Å^-1^ and 7.2 Å^-1^ in R and K spaces, corresponding to the Zn-O and Zn-Zn/O coordination, which was similar to the ZnO group. For Zn-foil, only one intensity at 7.5 Å^-1^ was observed, which attributed to Zn-Zn coordination. Above all, the results of EXAFS confirmed the Zn-O coordination in ZCE and the coordination was clearly.

### *In vitro* drug and Zn elements release

To evaluate pH-responsive drug release kinetics critical for antitumor efficacy, the pH-dependent release profiles of DOX and ICG were systematically characterized under physiologically relevant conditions (pH 7.4) and tumor microenvironment-mimicking conditions (pH 6.5). The release profiles of zinc element for ZnO and ZCE were also displayed. The entrapment efficiency (EE%) and drug loading capacity (DLC%) of DOX and ICG for different NPs were showed in Table S1. The release of ICG and DOX was feasible due to the fusion of EM film and gradual degradation of COF layer at pH 6.5 after endocytosis into cancer cells. In Figure S8, the cumulative release of DOX with laser irradiation at pH 7.4 and pH 6.5 were 12.9% and 46.2% respectively, suggesting selective release in tumor environment with little toxicity in normal physiological tissues. The release curve for ZDZIE was similar with ZDCIE and the dissolution rate was 63.5% and 12.6% at pH 6.5 and 7.4 (Figure S9). Interestingly, the release of DOX was slower at dark, with less than 13% released from the nanocarrier (Figure S10). For ICG, an accelerated release trend was clearly observed, as it was loaded into the external pores of ZIF-67/COF, promoting specific release in tumor tissues (Figures S11 and S12). The pH-responsive release of ICG and DOX was driven by the acid-sensitive degradation properties of ZIF-67 55, COF 56 and ZnO 57 in the tumor site (pH 6.5), respectively. Moreover, as shown in Figure S13, the premature release curves of DOX and ICG are gradually increased to only 7.84% and 3.82% for 2 h, which not occur the instant release condition in TME (pH=6.5) before being translocated into tumor cells. When transported into cancer cells, the cumulative release rates in ZCE of DOX and ICG are 28.06% and 17.55% during 4.5 h, suggesting the drug could be released adequately inside cells to exert antitumor effect. Elemental Zn is also gradually released from ZnO and ZCE in TME (Figure S14).

### Rapidly penetrated into cancer cell

**Figure 3:**
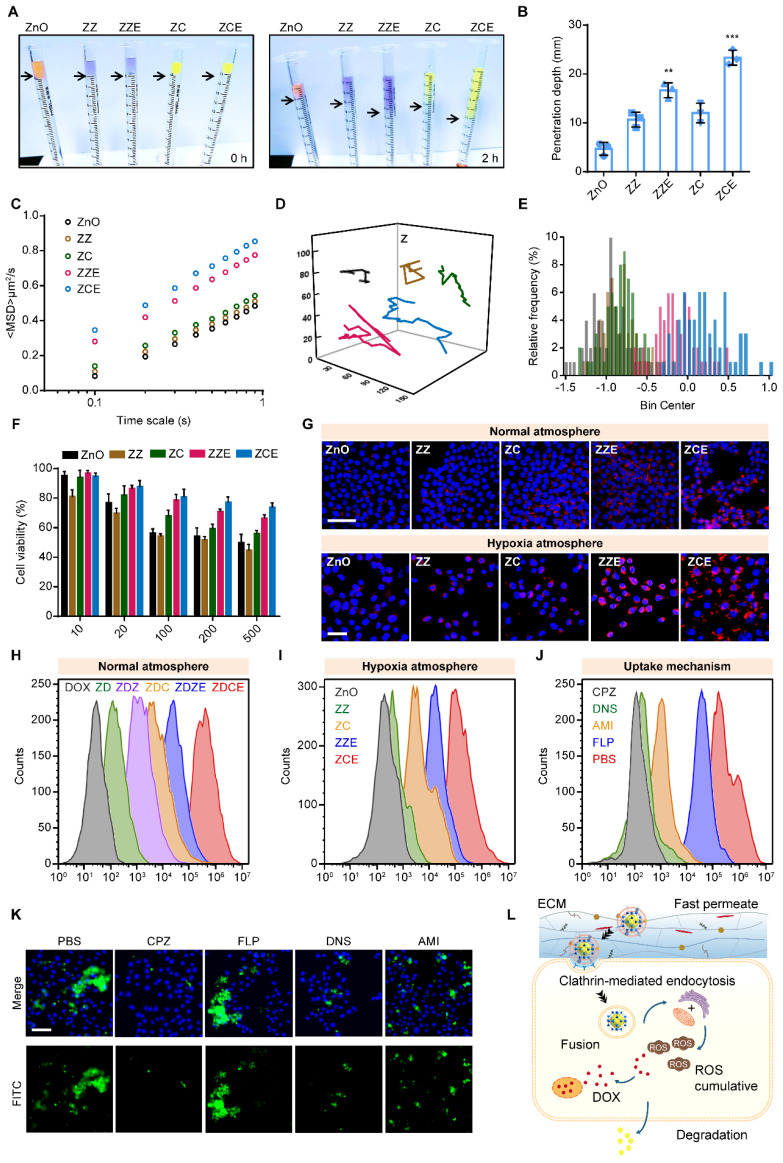
** Overcoming extracellular sequential barriers.** (A) The images and (B) quantitative data for depth of penetration in the stimulate ECM. (C) The values of MSD as a function of time scale. The data represent experiments in which 200 NPs were tracked. (D) The trajectories of NPs on a time scale of 1 s; The group was same with (C). (E) Distributions of the logarithmic D_eff_ values of NPs in the simulated ECM; The group was same with (C); (F) Cell viabilities of different NPs. (G) CLSM images of the internalized of NPs in 4T1 cells under normal and hypoxia atmosphere. Blue, nuclei stained with DAPI. Red, RITC-labeled NPs. Scale bars, 20 μm; Flow cytometry (FCM) analysis on the cell uptake at normal atmosphere (H), cell uptake at hypoxia atmosphere (I) and (J) cell uptake mechanism. (K) CLSM images of the endocytosis of NPs in 4T1 cells pretreated with CPZ, FLP, DNS and AMI. Scale bar, 50 μm. (L) Schematic illustration of transported route for NPs after endocytosis. The data are presented as means ± SD, n = 3, *p < 0.05, **p < 0.01 and ***p < 0.001.

When the NPs were transported into the tumor tissues to penetrate into the cellular, they encountered the ECM, a significant obstacle consisting of a cross-linked, gel-like viscous structure. In order to determine the penetration depth and trajectories of NPs, the multiparticle tracking (MPT) method was employed to investigate the diffusion capacities in a solution mimicking the tumor ECM (0.1% HEC). The penetration depth values of ZZ, ZC, ZZE and ZCE were approximately 2.3-, 2.6-, 3.5- and 5.0-fold higher than those of bare ZnO NPs respectively (Figures 3A and 3B). The mean square displacement (MSD) value of ZCE was 0.5-, 0.6-, 0.9-, 1.8-fold higher than that of ZZE, ZC, ZZ and ZnO (Figure 3C). The motion trails and MPT distribution also revealed that ZCE and ZZE moved randomly within the simulated ECM, whereas the other NPs were almost entrapped (Figures 3D and 3E). The increased penetration was contributed to EM facilitated deeper ECM penetration *via* the appropriate charge of membrane.

Firstly, before transported at cancer cells, 3-(4,5-dimethylthiazol-2-yl)-2,5-diphenyltetrazolium bromide (MTT) assay was performed to analyze the *in vitro* cytotoxicity of different NPs. The cell viability of non-drug-loaded NPs conducted at the concentrations ranging from 10 μg/mL to 500 μg/mL in the 4T1 cell lines, exhibited up to 50% cell viability, indicating low toxicity (Figure 3F). Among these, ZCE coated with COF and EM showed reduced toxicity due to the absence of Co^2+^. Once the NPs crossed the ECM and were transported at cancer cell surface, the cellular membranes acted as “gatekeepers”, regulating the internalization of NPs. For investigating the cellular uptake behavior, confocal laser scanning microscopy (CLSM) was applied, as shown in Figure 3G. ZZE and ZCE exhibited significantly higher cellular uptake in 4T1 cells, indicating that decorated EM was available to increase this process. In contrast, ZZ, ZC and ZnO NPs showed lower internalization, likely due to the ECM barriers and limited receptor availability on the cell membrane. In fact, the internalization efficiency of ZDCE was 10.7-, 4.5-, 2.7- and 1.5-fold than that of ZD, ZDZ, ZDC and ZDZE, indicating the superior “fusion” effect of EM and the better biocompatibility of COF (Figure S15A). Hypoxic tumor cells often exhibited altered metabolism, which could reduce the uptake of NPs. It was found that the endocytosis of ZCE was enhanced in the hypoxic tumor microenvironment. On the contrary, the internalization efficiency of ZnO was obviously slower attributed to the hypoxic atmosphere. Although ZC and ZCE showed comparable cellular uptake under normoxic conditions, ZCE exhibited better endocytosis efficacy under hypoxic conditions, which might result from both its “CAT-like” property and the assistance of myohemoglobin (Figure 3G). The quantitative data was in accordance with the results from the CLSM images (Figure S15B), supporting the conclusion that EM coating strategy increased the biocompatibility of NPs, thereby promoting internalization. Additionally, it demonstrated that COF could improve endocytosis efficiency in comparison with ZIF-67 in the real tumor tissue. Flow cytometry analysis of cellular internalization conditions were aligned with CLSM images, confirming the improved endocytosis ability of ZCE (Figures 3H-I). For further demonstrating the intracellular transport route of ZCE in the cancer cells, many kinds of endocytosis inhibitors such as chlorpromazine (CPZ), filipin (FLP), dynasore (DNS) and amiloride (AMI) were applied to verity the endocytosis pathway. As shown in Figures 3J and S16, CPZ and AMI groups were easily refrained, suggesting that the sphere-shaped ZCE was mainly taken up *via* a clathrin-mediated route as literature 58. Moreover, the membrane fusion was identified as another key endocytosis pathway due to the EM-coating for ZCE. Flow cytometry analysis of cell uptake mechanism confirming the endocytosis route of ZCE (Figure 3K). The complete transferred route was shown as follows (see Figure 3L): firstly, ZCE rapid traversed the ECM barrier with the assistance of EM coating; and then it undergoes endocytosis by clathrin-mediated route and membrane fusion, resulting in efficient intracellular absorption; finally, ZCE was moved toward mitochondria or endoplasmic reticulum, either through protein guidance or passive diffusion, to produce ROS.

### Mitochondria dysfunction induces Znproptosis

**Figure 4:**
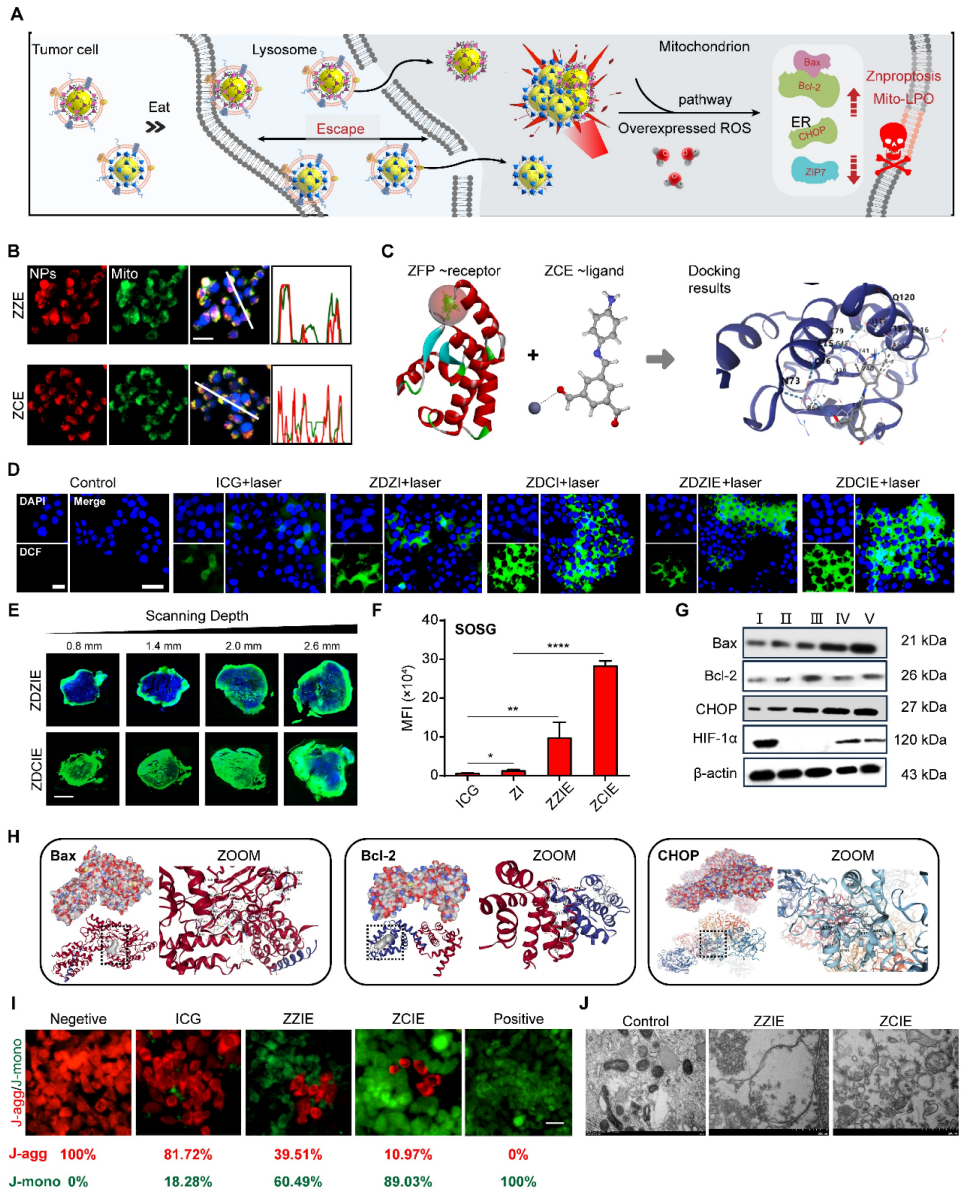
** Destroying of Mito.** (A) Scheme of trafficking route for NPs to kill cancer cells; (B) CLSM images of NPs (red) incubated with the mitochondria (green) of 4T1 cells. Scale bar, 5 μm; (C) Molecular docking between ZCE and zinc-finger proteins (ZFPs). (D) Intracellular ROS detected with DCFH-DA in 4T1 cells incubated with NPs for 4 h with laser irradiation. Scale bar, 20 μm; (E) ROS penetration detected with DCFH-DA into the tumor tissue under hypoxic conditions. Scale bar, 50 μm. (F) Qualification of ^1^O_2_ detected with SOSG incubated with NPs with laser irradiation in cancer cells. (G) Western blot analysis of proteins. I, ZnO; II, ZZ; III, ZC; IV, ZZE; V, ZCE. (H) Molecular docking between ZCE and Bax/Bcl-2/CHOP proteins. (I) The CLSM images of mitochondria membrane potential with JC-1 kit; Scale bar, 20 μm. (J) Bio-TEM of 4T1 cells after incubated with NPs (red arrow labels represent mitochondria). The data are presented as means ± SD, n = 3, *p < 0.05, **p < 0.01 and ***p < 0.001.

Similar to Curroptosis, it is predicted that the ROS-accumulation caused mitochondria dysfunction is the induction of Znproptosis, thus, the mitochondria dysfunction condition was detected. After the NPs uptake by fusion-dependent endocytosis, studies had demonstrated that membrane coated particles may prefer to aggregate in the Mito and endolysosomal regions to exert their destructive effects (Figure 4A).

As shown in Figure S17, when incubating for 2 h with NPs, the strong yellow fluorescence observed in colocalization studies indicated that the ZZ, ZC, ZZE and ZCE were trafficked to subcellular organelles, specifically the lysosomes. Then the preferred NPs including ZDCE could trigger the proton sponge effect, as evidenced by an increased red fluorescence signal. The internal flow of H^+^ and Cl^-^ *via* membrane ion channels led to the serve pH drop in the tumor micro-environment, contributing to the degradation of lysosomal components. Simultaneously, a similar “escape effect” was likely to occur at the tumor tissue level (Figure S18). For describing the comprehensive overview of intracellular activity, the number of NPs at subcellular organelle mitochondrial was detected. The results in Figure 4B showed that ZCE (red) and mitochondrial (green) were almost colocalization (yellow), indicating precise targeting of ZCE to mitochondrial. As reported, zinc-finger proteins (ZFPs) were in Mito, where most endogenous zinc was associated with metal-binding proteins and zinc storage proteins, resulting in extremely low levels of available zinc. At present, ZCE, containing zinc ions, could be recognized by ZFPs and formed a weak linkage, allowing them to anchor at the Mito (Figure 4C).

To confirm the ROS generation ability of NPs to destroy Mito, we investigated the effect on alleviating hypoxia and improving ROS production. The hypoxia indicator HIF-1α was selected to evaluate the oxygen production capacity of NPs. After incubating the cells with ZCE for 8 h, the minimal fluorescent intensity was observed, indicating that COF could release sufficient oxygen for ameliorating the hypoxic tumor micro-environment. Moreover, Pimonidazole HCl, another hypoxia marker, was also applied to determine the oxygen generation with results closely matching those of HIF-1α. After injection with the ZCE, the hypoxia-positive area decreased from 94.2% to 24.7% compared with that of ZnO group at cellular and tumor tissue level (Figure S19). These results clearly ensured that COF film NPs possessed excellent catalytic performance with H_2_O_2_ and significant potential for alleviating hypoxia in cancer cells. The ROS generation ability of NPs was investigated by CLSM using 2′,7′-dichlorofluorescein diacetate (DCFH-DA) as the indicator. Upon reacting with ROS, DCFH-DA was hydrolyzed and oxidized to 7′-dichlorofluorescein (DCF), which emitted a strong green fluorescence. The remarkable enhanced fluorescence intensity of DCF could be detected in ZDZIE and ZDCIE groups, suggesting the generation of ROS inside cells upon illumination (Figure 4D). It was noticed that a much stronger intensity of fluorescence in ZDCIE group was much stronger after laser irradiation, with levels 5.1-, 2.2-, 1.8-, 1.2-fold higher than those of ICG, ZDZI, ZDCI and ZDZIE, respectively (Figures S20-S21). The gating strategy used for flow cytometry analysis of cell uptake and ROS production was exhibited in Figure S22. With the laser irradiation, ROS was rapidly acquired from ZDCIE along time sequences (Figure S23). The penetrated depth of ROS into the overall tumor as shown in Figure 4E, mainly due to the intertumoral accumulation of ROS and the enhanced Znproptosis efficacy induced by ZDCIE. At the same time, the higher ROS released from ZDCIE with laser was observed compared to ICG, ZDZI and ZDZIE, ZDCIE at the tissue level (Figure S24). Another ROS index for ^1^O_2_, was evaluated using SOSG, and the results showed that ZCIE was 43.76-, 18.44-, 2.17-fold than ICG, ZI and ZZIE (Figure 4F). The decreased red fluorescence intensity of [Ru(dpp)_3_]Cl_2_ probe confirmed the higher production of ^1^O_2_ in ZCE group (Figure S25). The excellent ROS generation ability of ZDCIE might attribute to the superior energy transitions of Zn-COF complex, coupled with the self-production of oxygen. The expression of HIF-1α in the tumors treated with ZCE was also significantly lower than in the ZnO group, suggesting that large amounts of oxygen were constantly released and diffused throughout the tumor. At the same time, the apoptosis related proteins Bax, Bcl-2, and CHOP were activated immediately. In detailed, the expression of the pro-apoptotic protein Bax up-regressed, emergency protein CHOP was increased and anti-apoptotic protein Bcl-2 was down-regressed after incubated with ZCE, demonstrating the dysfunction of Mito and ER (Figure 4G). The Mito dysfunction mechanisms were shown in Figure 4H, suggesting that ZnO-COF possessed a positive ROS generation to kill cancer cells through Bax/Bcl-2/CHOP apoptosome. In detailed, ZCE-Bax/Bcl-2/CHOP compounds were stable with the high vina scores above -5.0. The specific therapy effect was evaluated *via* the endoplasmic reticulum (ER) stress response and the decline in mitochondrial membrane potential (MMP, ∆ψ). CHOP, a hallmark of ER stress, was highly expressed in cells treated with ZDCIE, as shown by the green fluorescence in Figure S26, indicating a strong ER stress phenomenon. Bax/Bcl-2 could control the release of cytochrome C, thereby triggering apoptosis. Furthermore, the depolarization of MMP was determined using the mitochondrial fluorescence probe JC-1. In general, after JC-1 staining, red fluorescence represented JC-1 aggregates (healthy mitochondria), while green fluorescence appeared as JC-1 monomers (depolarized mitochondria). Compared to the ICG laser irradiation group, a much stronger green fluorescence was observed in cells treated with ZCIE irradiation, owing to the enhanced MMP depolarization caused by ETC inhibition (Figure 4I). In Figure 4J, the clear dysfunctional Mito were observed which induced by ROS accumulation in Mito. Meanwhile, there was constant DOX release from ZDCIE under laser, “running” into the nuclei to provide chemotherapy by preventing the DNA repair process.

### The mechanism and signal pathway of Znproptosis

**Figure 5:**
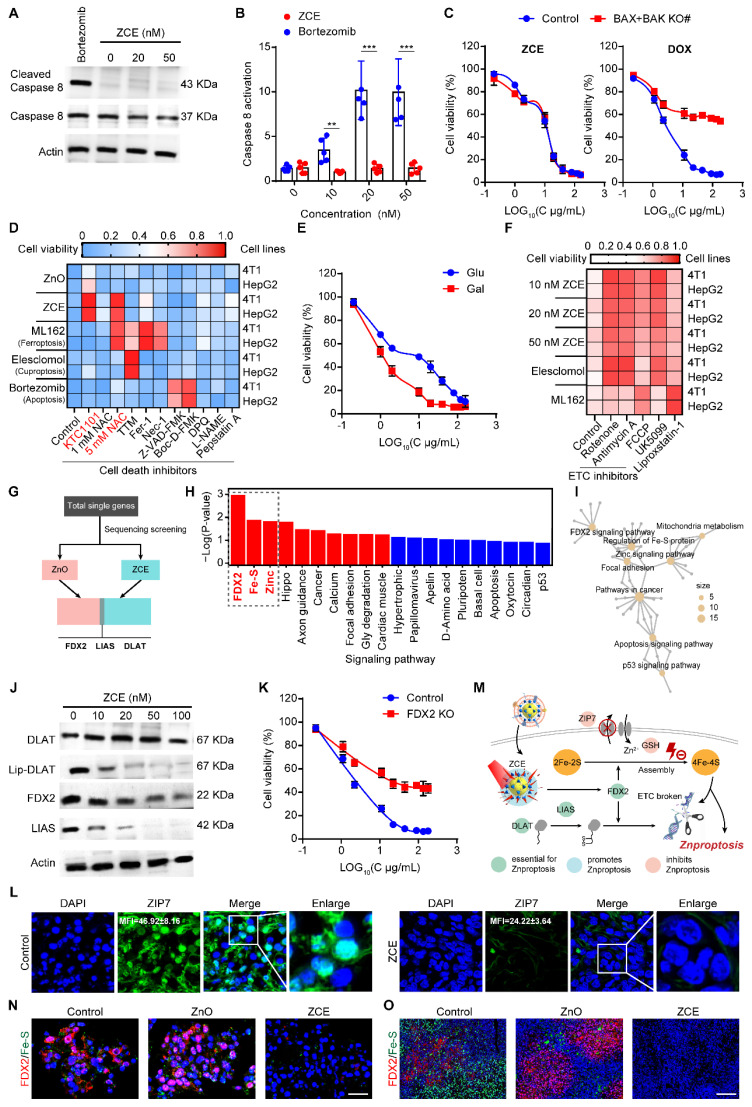
** Mechanisms of Znproptosis.** (A) Western blot analysis of ZCE or 10 mM bortezomib for 6 h and under irradiation for 5 min. (B) Caspase 8 cleavage in 4T1 cells after indicted treatments. (C) Cell viability of 4T1 cells or two clones with Bax/Bak deleted after treatment with ZCE and DOX under irradiation for 5 min. (D) Heatmap of viability of cells pretreated overnight with different inhibitors and then treated with samples for 72 h (average of three replicates). (E) Cell viability in media containing either glucose or galactose treated with ZCE under irradiation for 5 min. (F) Heatmap of viability of cells pretreated overnight with different Mito inhibitors and then treated with samples for 72 h (average of three replicates). (G) Genetic screening using RNA-sequence analysis in 4T1 cells of Znproptosis. (H) Bar plot enrichment of P-value and (I) network for signaling pathways. (J) Western blot analysis of ZCE for 6 h and under irradiation for 5 min. (L) Cell viability of 4T1 cells or two clones with FDX2 deleted after treatment with ZCE under irradiation for 5 min. (L) CLSM images of expression of ZIP-7 after incubated with ZCE. Scale bar, 10 μm, enlarge, 5 μm. (M) Schematic of mechanisms that promote Znproptosis. CLSM images of expression of FDX2 and Fe-S (4) in 4T1 cells, scale bar, 20 μm (N) and tumor tissues, scale bar, 50 μm. (O) after incubated with ZCE under NIR for 3 min under hypoxic condition. The data are presented as means ± SD, n = 3, *p < 0.05, **p < 0.01 and ***p < 0.001.

As expected, Mito-dysfunction was further leading to the Znprotposis and the mechanism of Znproptosis by which overload zinc ions and ROS activated was further explored. The previous reported the ZnO induced apoptosis cell death, but ZCE killed cancer cell did not involve the activation of caspase 8, the marker of apoptosis (Figures 5A-B). When the key proteins BAX and BAK were knocked out, the killing effect of ZCE was maintained, indicating the ZCE induced cancer cell death was distinct from apoptosis (Figure 5C). Moreover, the heatmap results related other cell death pathways, including Ferroptosis (ferrostatin-1), necroptosis (necrostatin-1), and oxidative stress (N-acetyl cysteine), were all not caused by ZCE (Figure 5D), suggesting a mechanism of ZCE was different from known cell death pathways. To further validate the zinc and reactive oxygen species (ROS)-dependent nature of Znproptosis, we quantitatively assessed the cytotoxic effects of zinc composite emulsion (ZCE) and zinc oxide (ZnO). The results demonstrated that Znproptosis induction required dual involvement of zinc ions and ROS generation, as evidenced by the dose-dependent cell death profiles (Figure 5D). (Figure 5D). It was observed that the pathways that mediate ZCE induced cell death was more reliant on pyruvate pathway than glycolysis (Figure 5E). The inhibitors of electron transport chain (ETC) complexes and pyruvate uptake (UK5099) in mitochondrial alleviated cell death, confirming the ZCE-mediated death pathway with great difference from Ferroptosis (Figure 5F). Particularly, FCCP, as the mitochondrial uncoupler to control adenosine triphosphate (ATP) production, had limited influence on Znproptosis, suggesting that Znproptosis was not related to ATP of mitochondrial respiration (Figure 5F). These results declared that Znproptosis damage the mitochondrial metabolism by the respiratory chain.

To identify the specific metabolic pathways that mediate Znproptosis, genetic screens to identify the key genes involved in Znproptosis. Considering the similar property between zinc and copper 59 and based on the key gene of Cuproptosis 60, FDX2, the homological protein of FDX1 was selected as the candidate key gene of Znproptosis. The RNA-sequence screening results confirmed the true prediction of FDX2 as the key gene of Znproptosis (Figure 5G). Moreover, the enrichment results suggested that the FDX2/LIAS/DLAT pathway was related to the Fe-S protein metabolism and could affect the energy production of Mito by disrupting respiratory chain (Figures 5H-I). As predicted, after ZCE treatment and NIR irradiation, the expressions of FDX2, Lip-DLAT and LIAS were both down-regulated, suggesting the Znproptosis was killing cell through interrupting the synthesized of iron-sulfur (Fe-S) proteins (Figure 5J). In Figure 5K, FDX2 KO rescued cells from Znproptosis, confirming the role of FDX2 as a key gene of Znproptosis. ZIP-7, as a zinc transported protein, could export the zinc to maintain the balance of zinc in Mito 61. As shown in Figure 5L, compared to control group, the green stained area was limited in ZCE group, indicating the expression of ZIP-7 was inhibited by Znproptosis. Taken together, the results supported a novel model whereby Znproptosis degraded Fe-S cluster proteins by FDX2 that results in proteotoxic stress and ultimately cell death (Figure 5M). As shown in Figures 5N-O, the expression levels of FDX2 and Fe-S are decreased significantly after treatment with ZCE under NIR for 3 min, confirming the mechanism of Znproptosis is regulating the FDX2 to interrupt the synthesized process of Fe-S (4). Additionally, in the Bio-TEM images, it is obvious that after Znproptosis induced by ZCE, the mitochondria are almost injury without cristae and occur swollen and brittle condition (Figure S27).

### Interrupt of cell cycle and Akt/mTOR pathway

**Figure 6:**
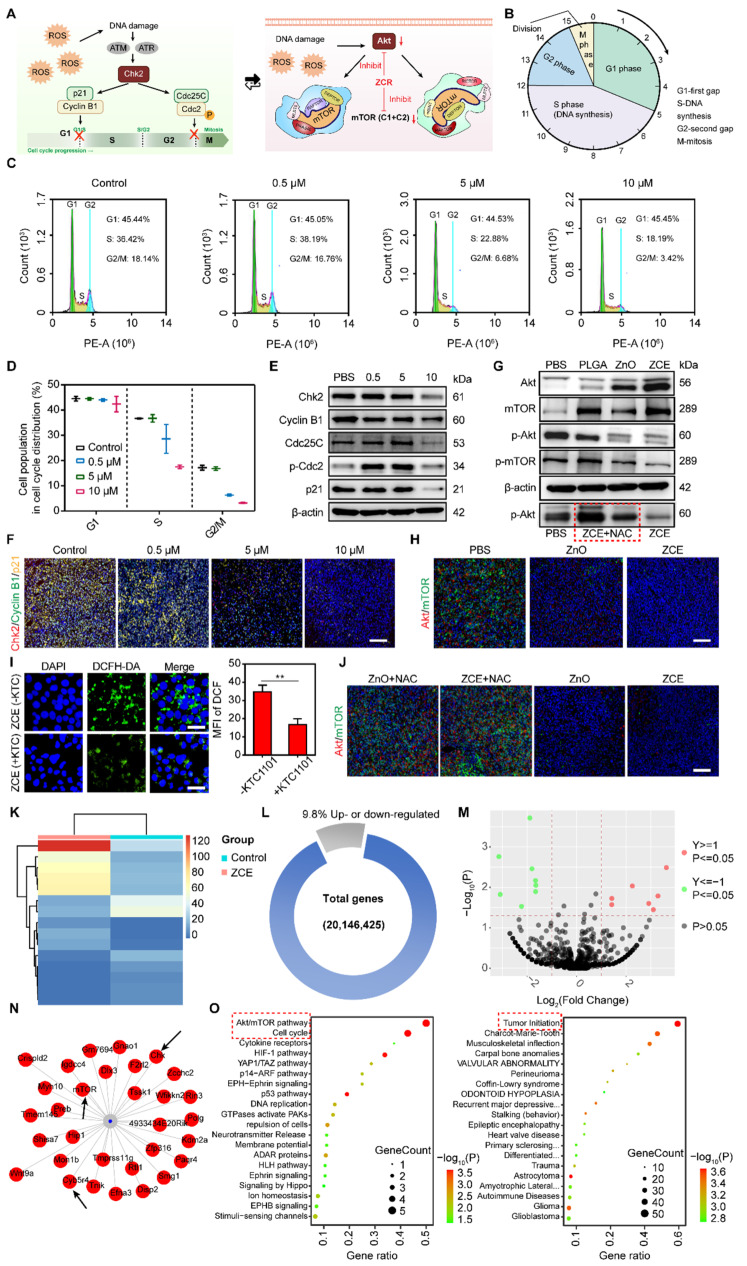
** The signal mechanism of Znproptosis.** (A) Schematic illustration of cell cycle and Akt/mTOR pathway. (B) The detailed route of cell cycle progress. (C) Flow cytometry analysis for cell cycle distribution of 4T1cells. (D) Quantitative data of cell population in cell cycle. WB analysis of cell cycle signal pathway (E) and Akt/mTOR pathway, in the red frame was ZCE added NAC to inhibit ROS. (G) Immunofluorescent staining of Chk2/Cyclin B1/p21 (F) and Akt/mTOR (H) of tumor tissues after treatment of nanocatalysts under hypoxic condition. Scale bar, 100 μm. (I) CLSM images of ROS production and quantitative data after incubated with KTC1101 under NIR for 5 min. Scale bar, 10 μm. (J) Immunofluorescent staining of Akt/mTOR of tumor tissues after treatment of nanocatalysts with/without NAC under hypoxic condition. Scale bar, 100 μm. (K) Heatmap of RNA-seq analysis of 4T1 cells incubated without or with ZCE. (L) The proportion of up- and down-regulated genes in 4T1 cells incubated with ZCE. (M) Volcano plots of differentially expressed genes in 4T1 cells incubated with ZCE. (N) The mRNA network map of expressed genes in 4T1 cells incubated with ZCE. (O) Gene ontology enrichment analysis of 4T1 cells incubated with ZCE. The data are presented as means ± SD, n = 3, *p < 0.05, **p < 0.01 and ***p < 0.001.

The inhibitory effects of Zn on cell cycle progression and cell viability were investigated in 4T1 cells. Akt/mTOR played an essential position in the regulation of cell cycle (Figure 6A). As reported, Zn could regulate the phases of the cell cycle before and after DNA synthesis (Figure 6B). Severe zinc accumulation can delay the transition of cells from the G1 phase to the S phase. Zinc can also regulate cell mitosis by affecting chromatin condensation during the post-DNA synthesis phase. Flow cytometry results suggested that ZCE could arrest cells in the G1/S and G2/M phases with an obvious concentration-dependent behavior (Figure 6C). The quantitative data described that S and G2/M phases were decreased by 50.1% and 81.1% respectively, at a concentration of 10 μM, inducing dysfunctional DNA repair (Figure 6D). Mechanistically, it was concluded that ZCE arrested cancer cell cycle *via* Chk2 signaling pathway. The activated Chk2 is a protein kinase to amplify the DNA damage signal from ATM.

When Cdc25C and Cdc2 are phosphorylated by Chk2, the G2 phase of cell cycle was arrested. Additionally, Chk2 also participated in the regulation of p21 to inhibit the transformation of Cyclin B1 to interrupt the development of G2/M phase. As shown as Figure 6E, it is noticed that ZCE with high concentration activated both the Chk2/Cdc25C/p-Cdc2 and Chk2/p21/Cyclin B1 signaling pathways, leading to cell cycle arrested at the G1/S and G2/M phases. The quantitative data of proteins were also verified the down-regulation of these signaling pathways in the cell cycle (Figure S28). The CLSM images were also suggested the improved expression of Chk2 and p21, which was consisted with WB results (Figure 6E and 6F). The arrest of G2/M phase also inhibited Akt/mTOR. Akt/mTOR signaling pathway was a crucial route to regulate the DNA replication during cell cycle progression and coordinate the activity of cyclin dependent kinases (CDKs). Moreover, Akt as a key protein also regulated the mTORC1 and mTORC2, which were formed by mTOR kinase. As shown in Figure 6G, significant increase in Akt and mTOR expression, along with a decrease in phosphorylated Akt (p-Akt) and phosphorylated mTOR (p-mTOR) were observed. The WB result in red frame showed that when ROS production was reduced by NAC, the expression of p-Akt increased, verifying the p-Akt levels respond to ROS (Figure 6G). The gray values analysis showed that ZCE up-regulated the Akt expression by 4.96-fold compared to ZnO and down-regulated the p-mTOR expression by 0.46-fold compared to ZnO. After incubation with NAC, p-Akt expression increased by 6.78-fold, consistent with WB images (Figure S29). Similarly, weak fluorescence was observed in CLSM images following with ZCE incubation, indicating the interruption of Akt/mTOR signaling pathway (Figure 6H). Further, the modulated mechanisms between Akt/mTOR and ROS production were also declared. In Figure 6I, when the key component of Akt pathway was inhibited by KTC1101, the ROS production reduced significantly to only 0.35-fold of that in ZCE group without KTC1101, indicating that the Akt pathway was modulated by ROS generated by ZCE. Moreover, Figure 6J showed that after incubated with the NAC to reduce ROS, expressions of Akt/mTOR increased substantially, suggesting promoted Akt/mTOR activity and confirming that ROS regulated the Akt/mTOR pathway. The results in Figure S30 further supported that high ROS production was associated with down-regulation of Akt/mTOR expression, confirming that ROS accumulation inhibited the Akt/mTOR pathway. Detailed genetic expression data indicated that the ROS generated by ZCE under irradiation down-regulated genes related to the Akt/mTOR pathway, including Akt family genes (Akt1, Akt2, Akt3 and Mtor) and regulatory genes involved in apoptosis and cell cycle (Bad and Cdc42) progression 62. In contrast, genetic expression in ZCE+NAC group was similar to that of PBS group, demonstrating that in the absence of ROS, regulation of the Akt/mTOR pathway was ineffective (Figure S31).

Moreover, the RNA sequencing-based analysis was determined to explore changes in the signal pathways at mRNA level following treatment with ZCE in 4T1 cells. The expression patterns, including up- or down- regulation of genes, were showed in Figure 5K. After incubated with ZCE under NIR, 4.9% of genes were up-regulated and 4.9% were down-regulated in 4T1 cells (Figure 6L). Top 15 up-/down- regulated genes, as induced by ZCE under NIR, were visualized in a volcano plot (Figure 6M) and mRNA network (Figure 6N). A clear difference in gene expression patterns between PBS and ZCE was observed, correlating with cancer cell behaviors such as cell proliferation, migration, invasion and, maturation. To further demonstrate the molecular signal pathways induced by ZCE, both the cell cycle signaling pathway and Akt/mTOR pathway were examined. The Akt/mTOR pathway is critical for various aspects of cell growth, survival, and regulation, both in physiological and pathological contexts. Amino acid like serine, which regulate this pathway, could be influenced by ZCE, acting as a regulator of amino acid metabolism. Other pathways related to induced by zinc ions and ROS overload including Ferroptosis pathway, hypoxia inducible factor 1 (HIF-1) signaling pathway, and p53 signaling pathway were also identified (Figure 6O). The intersection of Akt/mTOR and Chk2/Cdc/p21 pathways was driven by ROS accumulation of from ZCE, highlighting the nanocatalyst's peroxidase-like activity at gene level. Additionally, the ZCE regulated genes involved in tumor initiation, contributing to a potent antitumor effect through Znproptosis (Figures 6N-O).

### Immunomodulatory effect *in vivo*

**Figure 7:**
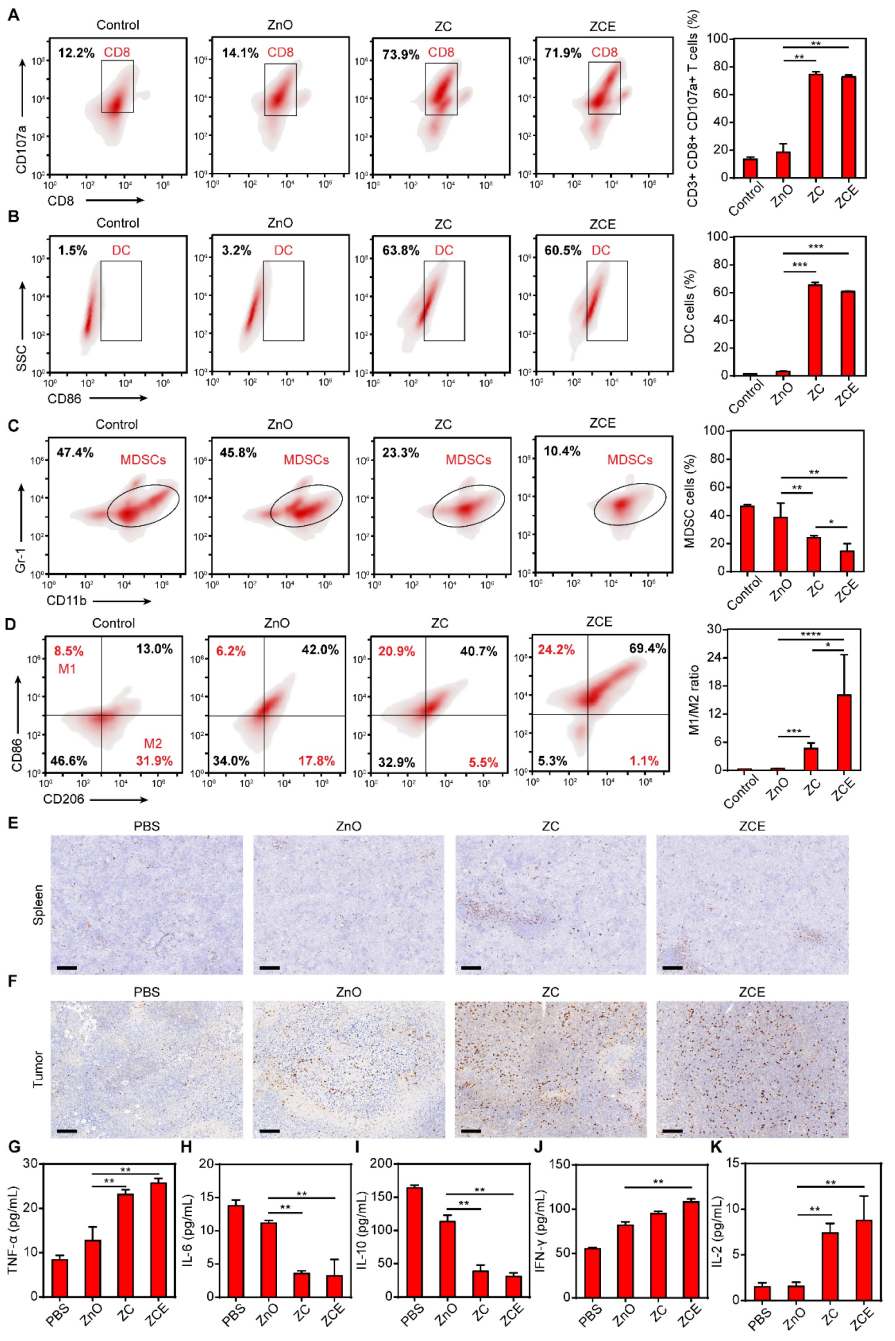
** Immunomodulatory effect *in vivo*.** Flow cytometry analysis and corresponding quantification of CD8^+^ T cells (CD8+CD107a, A), DC cells (CD86, B), MDSCs (Gr-1 CD11b, C) and M1/M2 (CD86 CD206, D) after NIR for 5 min. Immunohistochemistry staining of spleen (E) and tumors (F) for the subcutaneous tumor treatment model at the end of the treatment under hypoxic condition. Scale bar, 100 μm. Expression levels of immunity-related cytokines (including TNF-α, IL-6, IL-10, IFN-γ and IL-2) in tumor tissues (G-K) after different treatments under hypoxic condition. (n = 3, *p < 0.05, **p < 0.01, ***p < 0.001 and ****p < 0.0001).

Interestingly, the activation of Znproptosis in tumors also led to changes in the immune microenvironment. Thus, the immunomodulatory effect of nanocatalytic system was explored. First, the intrinsic immunogenicity of ZCE was verified by the increased proportion of CD8^+^ T cells 63, 64. The gating strategy used in the flow cytometry analysis for immune results was shown in Figure S32. ROS was released under NIR from ZCE sustainedly, leading to rapid activation and proliferation of CD8^+^ T cells (Figure 7A). MDSCs, a type of immunosuppressive cells that protects cancer cells by impairing T cell function were also examined. Dendritic cells (DCs) are a heterogeneous group of antigen-presenting innate immune cells that regulate adaptive immunity to kill cancer cells 65. The increased amounts of DC cells in ZCE group confirmed the higher antitumor ratios after NIR irradiation (Figure 7B). Before NIR irradiation, the increased amounts of CD8^+^ T cells and DC cells are all limited, indicating the Znproptosis-induced immunogenicity to anti-tumor need the trigger of NIR (Figure S33). The result in Figure 7C showed that ZCE as a nanocatalyst could continuously down-regulated the ratio of MDSCs thereby alleviating the immunosuppression within the TME. The M1/M2 proportion of TAMs was improvement obviously in ZC and ZCE groups under the Znproptosis-induced immune activation (Figure 7D). Immunohistochemical analysis further confirmed the increased infiltration of tumor-suppressive cytotoxic T cells (CD8^+^ T cells) under the ZCE-mediated augmented antitumor immunotherapy, compared to other groups (Figures 7E-F). Several cytokines, including TNF-α, IL-6, IL-10, IFN-γ, and IL-2 were selected as markers to assess immune cell function (Figures 7E-I). In ZCE with laser group, there was notable up-regulation of TNF-α and down-regulation of IL-6, suggesting immune cells activation. The decreased of IL-10 in tumor cells indicated a phenotypic shift in TAMs toward a tumor-suppressive state. Additionally, the increased expression of IFN-γ and IL-2 confirmed the increased activity of cytotoxic T cells. Moreover, as shown in Figure S34, the decreased expressions of promoting tumor survival immune factors (IL-4, IL-18, IL-1β and TGF-β) and the increased expressions of inhibiting cancer cell growth factors (IL-12 and IL-5) 66 after incubated with ZCE confirming the ZCE could regulated the immune system to antitumor with high efficacy. These findings collectively demonstrate the ability of the nanocatalytic system to regulate the immune response regulation at tumor site. The accumulated MitoROS could trigger Znproptosis and then led to the release of tumor-associated antigen (TAA) to improve the maturation of DC cells and activation of T cells for increasing the amounts of CD8^+^ T cells and pro inflammatory cytokines and immunogenic components from tumor cells, accelerating antitumor immune responses (Figure S35). Zinc ions in ZCE played a critical role in this immunomodulatory effect. Beyond causing MitoROS accumulation, which led to elevated levels of interferon and inflammatory cytokines, zinc ions also triggered the secretion of pro-inflammatory cytokines by activating intracellular metal ions' channels, such as potassium and calcium channels, further regulating the signal pathways for anti-tumor immunotherapy 67, 68.

### Immune phagocytosis avoidance effectively

**Figure 8:**
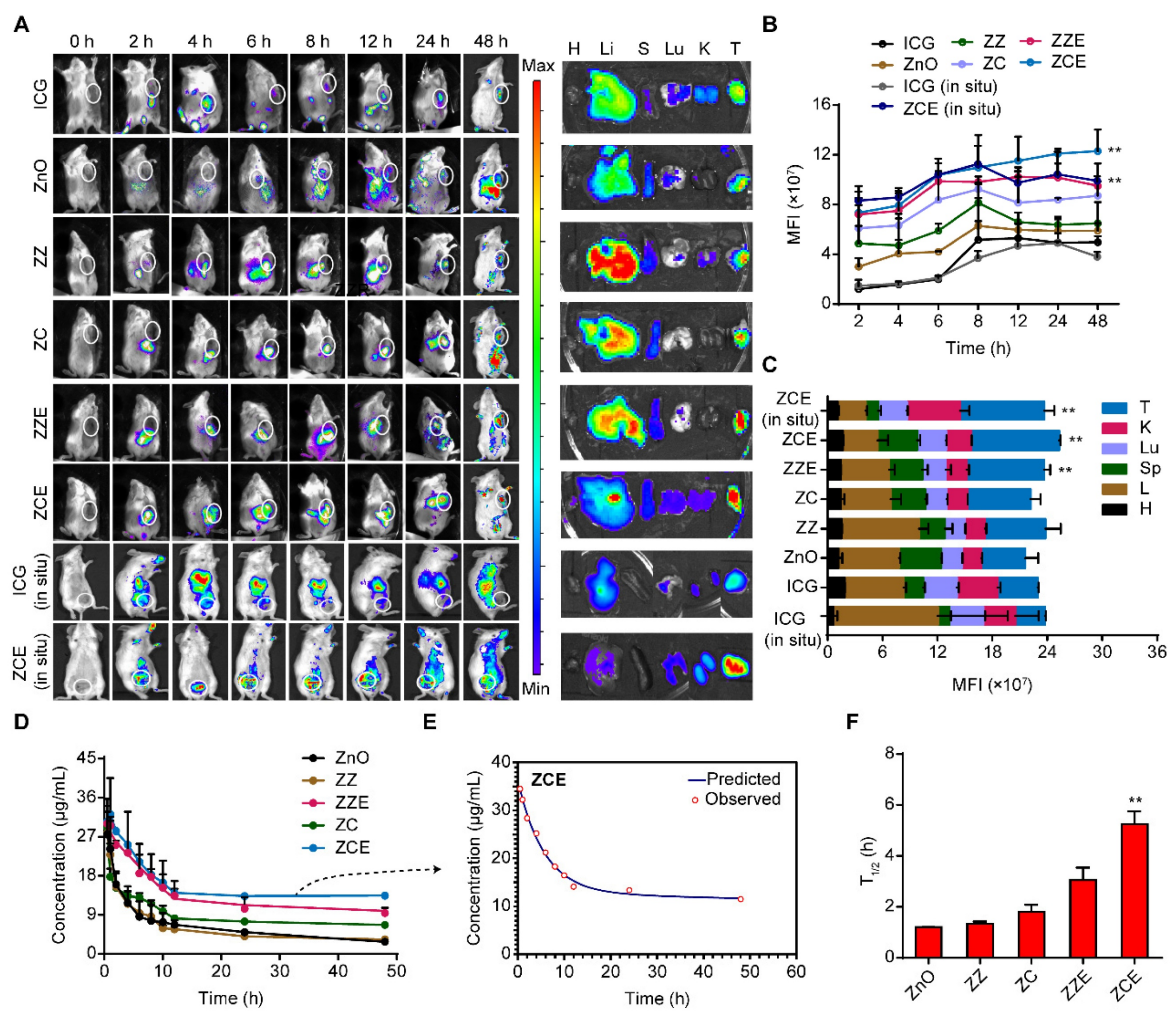
** Particles distribution evaluation in tissues.** (A) Distribution of the nanoparticles in Balb/c mice obtained *via in vivo* imaging and *ex vivo* images of major organs and tumors 48 h post-injection (The white circles are tumor sites). Scale bar: 5 mm; (B) Quantitative analysis of *in vivo* imaging at different time points (Inset images are the initial fluorescence intensity before injected in mice for ICG, ZnO, ZZ, ZC, ZZE and ZCE groups (from left to right)); (C) Quantitative analysis of major organs and tumors 48 h post-injection;(D) Residual contents of nanoparticles in the body after tail vein injection; (E) Predicted curve of ZCE in the body after tail vein injection compared with the observed results; (F) The half-time of nanoparticles in the body after tail vein injection. The data are presented as means ± SD, n = 3, *p < 0.05, **p < 0.01 and ***p < 0.001.

Since the distribution of NPs *in vivo* following intravenous injection was still unclear, we assessed their biodistribution in Balb/c mice with 4T1 cell-induced tumor. Figure 8A showed the NPs distribution behavior in tissues systemically. The fluorescent signals revealed that all the NPs smaller than 500 nm initially accumulated in liver after 8 h due to the circulation through the hepatic vein following injection. By 48 h, both ZZE and ZCE showed higher accumulation at tumor site, whereas other NPs primarily accumulated in liver, spleen and kidneys for metabolism. Additionally, according to the ICP-MS analysis, the Zn elements also distributed in tumor site of ZCE group, which in consist with NPs (Figure S36). Fluorescent imaging of major organs and tumor tissues from tumor bearing mice verified that ZZE and ZCE had higher aggregation at tumor site *via* EPR effect (Figure 8A). The fluorescent intensity of ZZE and ZCE at tumor site was 1.7- and 2.8-fold higher than that of their non-film coated counterparts (Figure 8B). The detailed distributions of ZZE and ZCE in each organ and tumor were exhibited as Figure 8C with a broad diffusion towards tumor and anchorage, which also was in consistent with Figure 8A. These data further highlighted the excellent performance of ZZE and ZCE in terms of deep tumor permeation. In addition, in the orthotopic breast cancer mice models, ZCE also could arrive at orthotopic tumor site by EPR effect and assistance of EM (Figures 8A-C), confirming its great tumor targeting ability and clinical value.

We then examined the blood concentration of different nanoparticles and verified that EM coating significantly extends their circulation time. In contrast to uncoated nanoparticles, which exhibited limited diffusivity and shorter circulation times, EM-coated ZZE and ZCE displayed improved stability and longer “anchorage” times* in vivo,* with ZCE showing the most optimal results (Figures 8D-E). The results showed that the T_1/2_ of ZCE was 4.4-, 3.9-, 2.9-, and 1.7-fold longer than that of ZnO, ZZ, ZC and ZZE respectively, denoting the superior biocompatibility of dual-film coated ZnO (Figure 8F). Simultaneously, the lower plasma absorption of ZCE (Figures S37-S38) suggested its stability in general circulation. The extended “transit” and “anchorage” times of ZCE denoted that the EM and COF layers further prolonged its activity, allowing more time to damage the ER and mitochondria of cancer cells.

### Highly Znproptosis rate of nanocatalytic system

**Figure 9:**
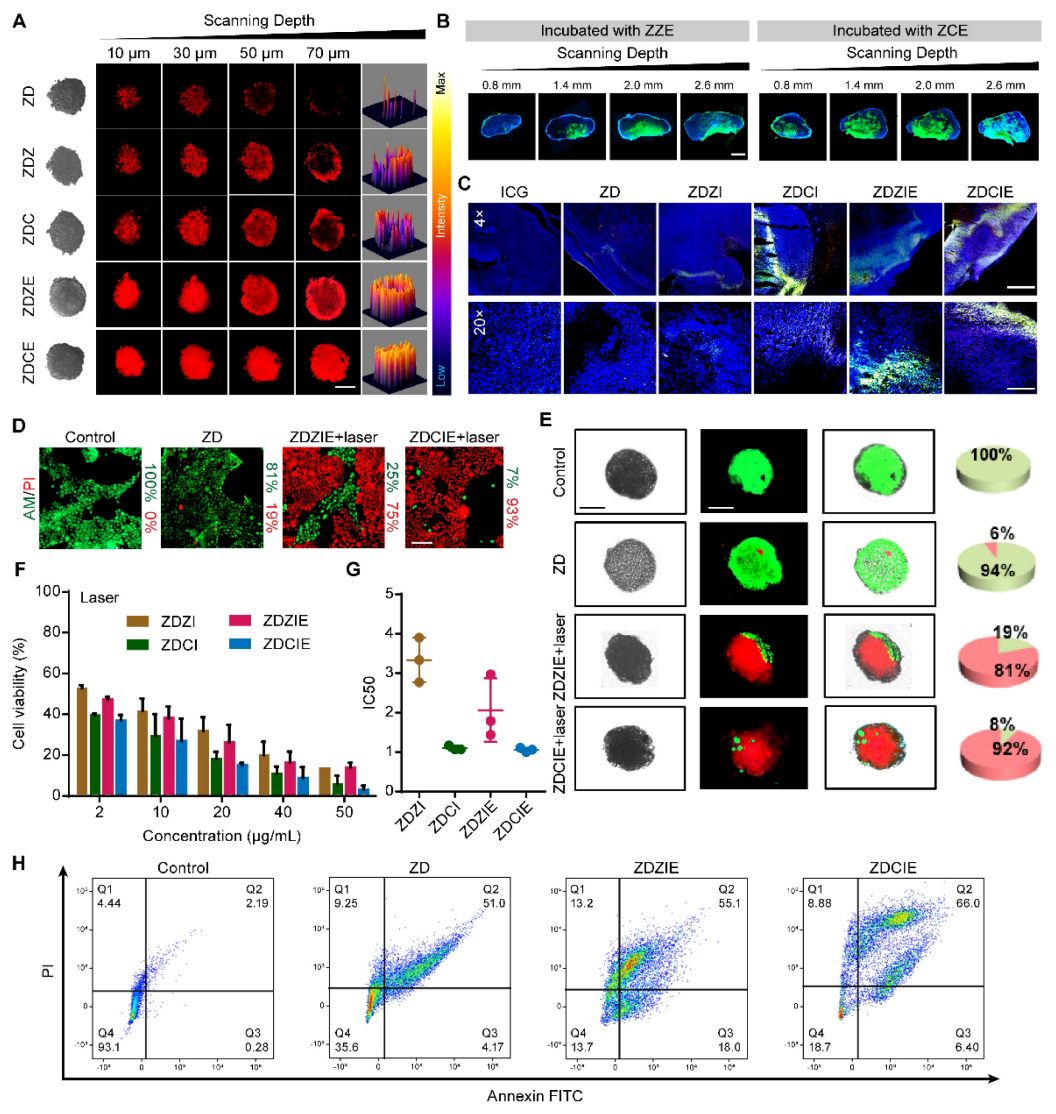
** Penetration capacity and antitumor effect studies.** (A) CLSM Z-stack images of NPs penetration and the 3D-renderings into the multicellular spheroids of 4T1 for 20 µm intervals. Scale bar: 100 μm; (B) CLSM images show the distribution of NPs-FITC labeled (green) in 4T1 tumors under hypoxic condition. The tumors were sliced and imaged with a 10× objective. The nuclei were stained with DAPI (blue). Scale bar: 2 mm (n=5); (C) CLSM images of intratumor distribution of DOX (red) and ICG (green) for 2 h after injection under hypoxic condition. Cell nuclei of tumor slice were stained with DAPI (blue). Scale bars, 200 μm; (D) CLSM images of calcein AM and propidium iodide (PI) staining 4T1 cells treated with NPs and then irradiation for 5 min. Live/dead cells were green/red. Scale bar, 20 μm. (E) CLSM images of calcein AM and propidium iodide (PI) staining 3D spheroids of 4T1 treated with NPs and then irradiation for 5 min. Live/dead cells were green/red. Scale bar is 150 μm. (F) Cell viability in 4T1 cells with laser. (G) The IC50 values of 4T1 cells with laser. (H) Flow cytometric analysis in 4T1 cells with irradiation for 5 min. The data are presented as means ± SD, n = 3, *p < 0.05, **p < 0.01 and ***p < 0.001.

Previously, we have proved that EM facilitated NPs were enabled to permeate meshwork, but it remained unclear whether the nanoparticles had a superior transport and trafficking capability in tumor tissue to generate the ROS for Znproptosis. Thus, we examined the permeated capacity of NPs in 3D tumor spheroids, simulating the tumor environment, and labeled the NPs with DOX.ZDCE penetrated to a depth of approximately 70 μm after 2 h, while the penetration depths of the other NPs were less than 50 μm, with the ZnO group reaching less than 30 μm. The 3D renderings clearly showed similar distributions of the NPs in 3Dtumor model (Figure 9A). The penetration abilities of in FITC-labeled ZZE and ZCE were monitored by CLSM (Figure 9B). Interestingly, ZCE group possessed abundant green signals in the tumor core while ZZE signals were concentrated at the tumor edges. At the scanning depth of 2.6 mm, the fluorescence intensity of ZCE was approximately 2.0-fold higher than that of ZZE. In addition, CLSM images of tumor slices indicated that DOX and ICG from ZDCIE had effective penetration, which was about 7.1-, 2.3-, 1.6- and 1.2-fold higher than ZD, ZDZI, ZDCI and ZDZIE respectively (Figure 9C). The superior penetration ability of ZDCE caused its enhanced apoptosis effects. The cells treated with ZDCIE and subjected to laser irradiation displayed intense red fluorescence and negligible green fluorescence, as confirmed by calcein AM/PI double staining, suggesting that approximately 93% cancer cells had died, ensuring the excellent Znproptosis induced by zinc ions and ROS overload from ZDCIE (Figure 9D). As shown in Figure 9E, 3D spheroids incubated with ZDCIE and irradiated for 5 min were destroyed by 92%, a rate 15.3- and 1.1-fold higher than in ZD and ZDZIE groups, respectively. While these results were based on 3D spheroids, it is essential to verify the actual cell death rate in tumor tissue to confirm these findings. These results suggested that ZCE was more effectively internalized into tumor tissues, resulting in a powerful and sustained Znproptosis effect induced by zinc ions and ROS overload. Similar results could be found from cell viability of ICG and DOX loaded NPs. Without laser irradiation, cell survival exceeded 50%, even with DOX concentrations as high as at 10 μg/mL, indicating slow cell death (Figure S39). However, when the laser switched on, ZDCIE (with DOX at 10 μg/mL) killed 70% of cancer cells, proving the excellent Znproptosis inducing effects driven by zinc ions and ROS overload (Figures 9F-G). This potent antitumor effect was also observed in other cancer cell lines, such as Hela and HepG-2, where the effectiveness increased with higher concentrations (Figure S40). The flow cytometry results further confirmed the induction of Znproptosis (Figure 9H). These results demonstrated that stable ZDCIE showed exceptional tumor accumulation throughout tumor tissue, driven by its ability to induce Znproptosis.

### High anti-tumor efficacy induced by Znproptosis

The significant antitumor efficacy was directly linked to Znproptosis mechanism. Building on the promising *in vitro* results, we next investigated the therapeutic efficacy of NPs on 4T1 tumor-bearing mice for ectopic and *in situ* location. Before intravenous injection, the hemocompatibility was determined using a hemolysis assay. The results showed that the hemolysis ratios for ZC and ZCE remained below 10% even at concentrations as high as 800 μg/mL, highlighting their excellent biocompatibility *in vivo* (Figures S41 and S42). Additionally, cell viability for ZDZIE and ZDCIE decreased to below 20% in a hypoxic environment under NIR irradiation (Figure S43), suggesting the outstanding stability in leading to apoptosis. The absorbance changes of ROS probes as DPBF and TMB were observed after incubation with ZZ, ZC, ZZE, and ZCE in the mimic tumor site with GSH, demonstrating that nanocatalysts (ZZ, ZC, ZZE and ZCE) could interact with GSH and then trigger mitochondrial lipid peroxidation (Figures S44 and S45).

**Figure 10:**
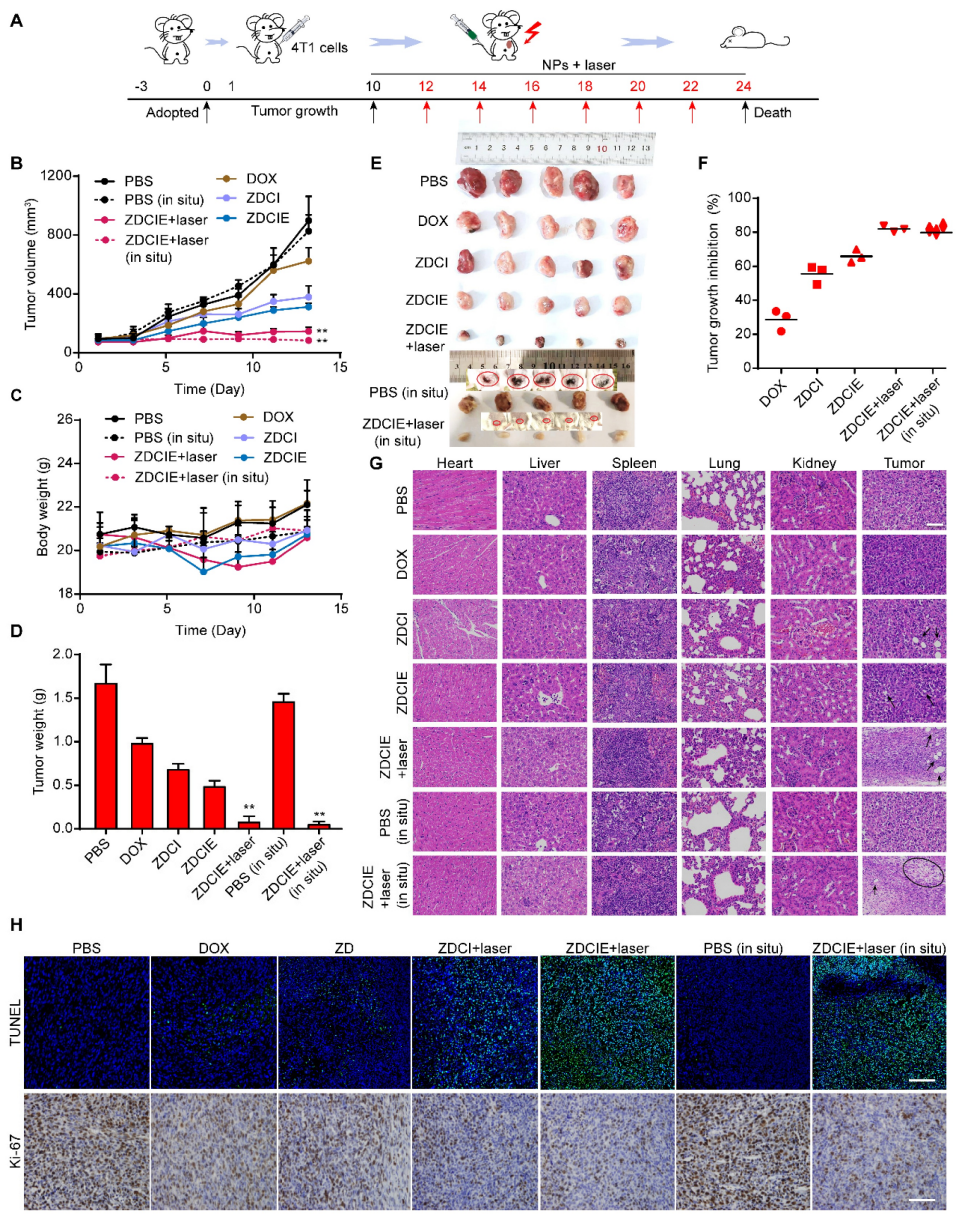
***In vivo* tumor growth inhibition study.** (A) Scheme of administration to inhibit the tumor growth; (B) Tumor growth volumes of mice after treatment with nanoparticles examined 14 days after the injection, mean ± SD; (C) Changes in the body weight of the mice after 14-day injection with different NPs *via* tail veins, mean ± SD; (D) The tumor weight in different groups after 14 days, mean ± SD; (E) Representative tumor images, Scale bar: 4 mm; (F)Tumor growth inhibition rate of mice after treatment with nanoparticles examined 14 days after the injection, mean ± SD; (G)The H&E staining of organs and tumors of the different nanoparticle groups under hypoxic condition. Scale bar: 100 μm. (H) TUNEL and Ki-67 analysis of different treatments under hypoxic condition. Scale bar: 20 μm. ns, *p* > 0.05; *p < 0.05; **p < 0.01, and ***p < 0.001. (n = 5).

Then the tumor inhibition effect was systematically evaluated in tumor-bearing Balb/c mice. When the tumor volumes reached about 100 mm^3^, the tumor-bearing mice were administered DOX/ICG-loaded nanoparticles (10 mg/kg) *via* tail vein injection every two days. The mice were randomly divided into nine groups: (1) control, (2) free DOX, (3) ZnO@DOX (ZD) (4) ZDCI NPs (5) ZDCI+laser, (6) ZDCIE, (7) ZDCIE+laser, (8) control *in situ*, and (9) ZDCIE+laser *in situ*. Groups 5, 7 and 9 were irradiated with an 808 nm laser for 5 min (2 W/cm^2^) after 12 h injection. Tumor volumes and body weights were recorded every other day (Figure 10A). After 14 days of treatment, as shown in Figures 10B-E and S46, the tumor volumes in ZDCIE+laser group increased slowly, and the mean tumor weights were significantly reduced, indicating a strong suppressive effect on tumor growth, due to the enhanced apoptosis induced by the COF. On the contrary, the DOX, ZD, ZDCI, ZDCI+laser and ZDCIE groups possessed the moderate inhibition efficacy with higher tumor volumes and weights, reflecting the insufficient antitumor effect of chemotherapy alone. Notably, ZDCIE+laser group exhibited superior tumor growth inhibition compared to the non-irradiated group, demonstrating that hypoxia triggered continuous ROS release. Moreover, ZDCIE+laser group exhibited more pronounced inhibition of tumor growth than ZDCI+laser group, due to the synergistic effects of EM and Znproptosis (Figure S46). Based on the tumor weights variation, the relative inhibition rate in the ZDCIE+laser group was calculated to be 83.8%, which is 1.6- and 1.1-fold higher than that of chemotherapy alone (ZD) and ZDCI groups, respectively (Figure 10F).

Hematoxylin and eosin (H&E) staining of organs and tumors after 14 days of treatment was conducted to evaluate the toxicity of the nanoparticles. Compared with the minimal damage observed in other groups (PBS, DOX, ZD, ZDCI, ZDCI+laser, ZDCIE and ZDCIE+laser groups for ectopic tumor and PBS, ZDCIE+laser groups for *in situ* tumor), severe damage in cancer cells was evident in the group treated with ZDCIE under laser irradiation. The H&E staining and TUNEL images of the tumor demonstrated that the ZDCIE nanoplatform, functioning as both a ROS and chemotherapeutics carriers, successfully permeated deep into tumor tissue, alleviated hypoxia, and remarkably improved antitumor therapeutic efficacy (Figure 10H).

In the orthotopic breast cancer mice models, the antitumor effect of ZDCIE+laser group was also great and the tumor inhibition rate was 83.2%, which was similar to tumor xenograft mouse models, simultaneously without toxicity of main organs (Figure 10). Furthermore, the excellent tumor inhibition rates observed in Hela and HepG2 tumor-bearing mice, which were 97.17% and 96.39% respectively, highlight the broad anti-tumor potential of the nanocatalytic system, alongside its good biosafety profile (Figure S47). At the same time, there was no apparent inflammation or pathological hurts were found in main organs of mice in all five groups, validating the safety of nanoplatform (Figure 10G). Additionally, body weight remained stable for mice treated with ZZE and ZCE, compared to PBS group, further assuring the low toxicity of NPs (Figure S48). Liver and kidney function markers, including BUN, T-CHO, AST and ALT, showed no significant differences between ZZE, ZCE and PBS groups, indicating the superior safety of NPs (Figure S49). The ZCE was also obtained long-term biocompatibility without triggering adverse immune responses over 3 months, supporting its potential for further application (Figure S50). The ROS generated by irradiated ZDCIE led to enhanced organelle apoptosis, contributing to the system's improved anti-tumor efficacy. At the same time, the nanocatalysts remained intact and stable in the bloodstream, with limited protein corona formation during the initial treatment period (Figure S51).

## Conclusions

In summary, the multifunctional nanocatalytic system was fabricated as a “permanent motivator” for generating ROS with cascade amplification effect (CAE), arresting cell cycle progression by inhibiting Akt/mTOR signaling pathway, and driving Znproptosis. Specifically, upon entering the cancer cells either through interactions between Zn and ZFPs directing them toward Mito, ZCE disrupted the preparation of Fe-S protein in mitochondrial electron transport chain (ETC) through down-regulating the expression of FDX2/LIAS and ZIP7 to activate Znproptosis. It also interrupted the G1/S and G2/M phases by regulating the expression of Chk2/Cdc25C/Cdc2 and Chk2/p21/Cyclin B1. COF served two key roles: first, in combination with Zn^2+^, it maximized ROS (·OH and^ 1^O_2_) production and maintained excited triplet state (ETS) for a long time by electronic transfer; second, when paired with EM, it intelligently self-generates oxygen, providing ample materials for ROS production. Zinc ions contributed to immunomodulatory effects for anti-tumor immunotherapy by triggering the secretion of pro-inflammatory cytokines. The “camouflage” layer of EM prominently enhanced penetration, endocytosis and tumor immune evasion. Additionally, the platform could intelligently control the drug releasing through its pH-sensitive properties. Consequently, this nanocatalytic system, with its ability to induce Znproptosis, holds promising potential for the treatment of solid tumors in the future.

## Supplementary Material

Supplementary methods, figures and tables.

## Figures and Tables

**Table 1:** FTIR vibrational frequency and assignments of complexes ZnO, ZZ and ZC.

Wavelength (nm)^ a^				Assignment ^b^	
ZnO	ZZ	ZC			
467 w	750 s	683 vw	ν(C-C), ν(C-N), δ(C-H)		
683 vw	967 m	850 m	δ(O-H), γ(C-H)		
	1150 s	1283 s	Ring breathing		
1400 w	1333 vs	1417 s	δ(C-H)		
	1450 vs	1683 vs	ν(Zn-O)		
1617 m	1617 w	2933 m	-		
3450 vs	2950 vw	3433 s	ν(C-H)		
					

a: vs, very strong; s, strong; m, medium; w, weak; vw, very weak; b: ν, stretch; δ, in-plane-bend; γ, out-plane-bend.
