# Supplementary material for: Nanocatalytic system releases overloaded zinc ions and ROS to induce Znproptosis and interrupt cell cycle through inhibiting Akt/mTOR pathway
Source: Theranostics. 2025 Mar 24;15(10):4734–62. doi: 10.7150/thno.107025 (PMC11984402; doi:10.7150/thno.107025)
Supplement: Supplementary file 1 — Supplementary methods, figures and tables. [file thnov15p4734s1.pdf]

Supporting Information

Nanocatalytic system releases overloaded zinc ions and ROS to induce Znproptosis and interrupt cell cycle through inhibiting Akt/mTOR pathway

Zi-Yue Xi [a], Chuan-Yong Fan [a], Ying-Ying Jiang [a], Xin-Ran Xi [a], Gan-Yu Nie [a], Shuang Zhu [a], Jun-Jie Zhang [a], Lu Xu [a,*]

a School of Pharmacy, Shenyang Pharmaceutical University, Shenyang, 110016, China

* Corresponding author E-mail address: xulu@syphu.edu.cn (L. Xu).

1. Supporting methods

2. Supporting results

(1) Supporting Table

(2) Supporting Figures

1. Supporting methods

1.1. Purification of RBCM

The plasma from rats was mixed with 1 mM EDTA (10 mL) and then centrifuge (1500 rpm) for 5 min at 4 °C and washed by distilled water. Afterward, the precipitates were suspended with 1 mM EDTA and added into 3.8 mL distilled water and PBS 7.4 (20×, 200 μL). Then, the solutions were centrifuge for 10 min at 4 °C (16000 g) and washed by distilled water. The obtained precipitates were RBCMs.

1.2. Analysis of DOX and ICG entrapment efficiency

For ZD, ZnO and DOX were dispersed in 5 mL phosphate buffer solution (PBS, pH 7.4) and stirred for 24 h at 25 °C, then the precipitates were collected by centrifuging and removed the extra drug with PBS. Then the NPs was drying by vacuum drying to obtain ZDs.

For ZDZI and ZDCI, ZDZ, ZDC and ICG were dispersed in 5 mL phosphate buffer solution (PBS, pH 7.4) and stirred for 24 h at 25 °C, the precipitates were collected by centrifuging and removed the extra drug with PBS. Then the NPs was drying by vacuum drying to obtain ZDZI and ZDCI.

The entrapment efficiency (EE) and drug loading capacity (DLC) were calculated by equations (1) and (2).

$$EE(\%)=\frac{M_d}{M_N}\times 100\% \quad (1)$$

$$DLC(\%)=\frac{M_d}{(M_t+M_N)}\times 100\% \quad (2)$$

The M_d was the mass of drug in NPs, M_N was the mass of total NPs, M_t was the mass of total drug.

1.3. Size and Polydispersity (PDI) Stability analysis

The nanocatalysts (1 mg) was dissolved in PBS 7.4 and plasma (v/v 1:1) solutions, and then measuring the size and polydispersity of particles at 0, 5, 10, 20, 40, 60, 80, 100 h by Zetasizer NanoZS90 (Malvern Instruments Ltd., UK).

2. Supporting results

(1) Supporting Table

Table S1. DLC% and EE% values of NPs loaded with DOX, ICG and DOX/ICG

Formulation	ZD	ZZIE	ZCIE	ZDZIE	ZDCIE
DLC $_{Dox}$ (%)	21.24	-	-	20.70	20.43
EE $_{Dox}$ (%)	92.02	-	-	89.69	88.54
DLC $_{ICG}$ (%)	-	20.93	19.01	21.61	21.29
EE $_{ICG}$ (%)	-	90.70	82.38	93.64	91.83

Table S2. EXAFS fitting parameters at the Zn *K*-edge for various samples.

Sample	Shell	CN[a]	R(Å)[b]	σ^2(Å^2)[c]	ΔE_0(eV)[d]	R-range/Å	R factor
Zn foil	Zn-Zn	6*	2.65±0.01	0.0112±0.0007	1.5±0.9	1.0-3.0	0.0061
	Zn-Zn	6*	2.82±0.01	0.0250±0.0048	2.7±1.3		
ZnO	Zn-O	4.0±0.4	1.97±0.01	0.0036±0.0012	5.9±0.6	1.0-3.7	0.0061
	Zn-Zn	17.4±2.2	3.24±0.01	0.0106±0.0010	4.7±0.3		
	Zn-O	18.4±1.7	3.79±0.01				
ZCE	Zn-O	4.2±0.3	1.97±0.01	0.0040±0.0010	4.8±0.5	1.2-3.7	0.0053
	Zn-Zn	14.6±2.0	3.24±0.01	0.0117±0.0012	4.4±0.3		
	Zn-O	16.2±1.5	3.80±0.01				

(2) Supporting Figures

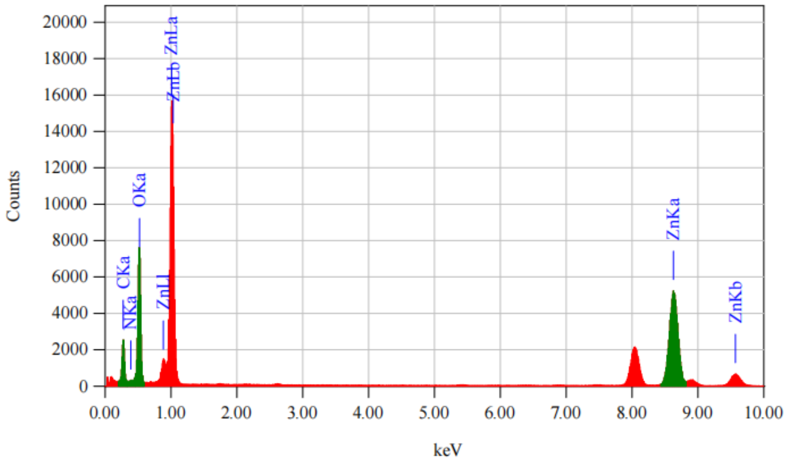

Figure S1. The EDS spectrum of ZCE.

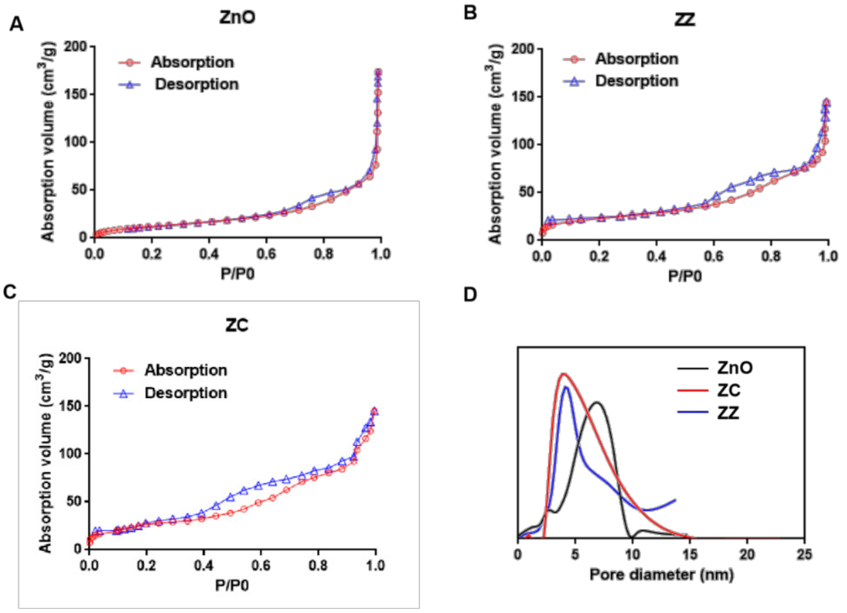

Figure S2. The Nitrogen adsorption/desorption curve of (A) ZnO; (B)ZnO@ZIF-67; (C) ZnO@COF and (D) the pore size of NPs.

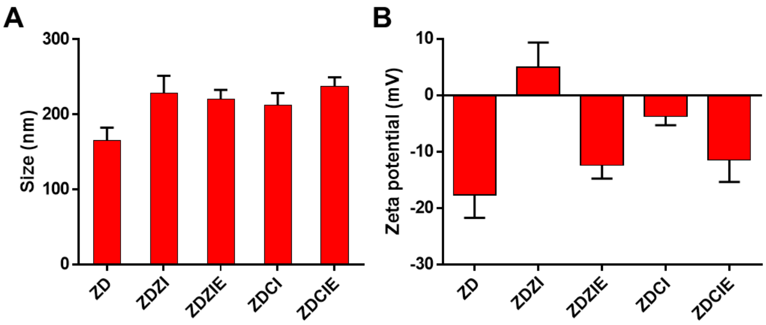

Figure S3. The sizes and zeta potentials of the drug-loaded nanoparticles.

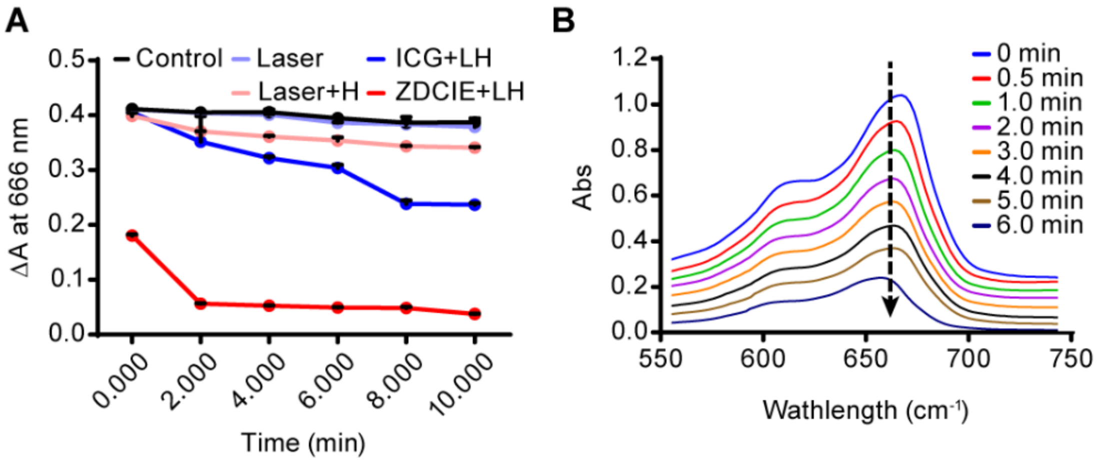

Figure S4. (A) Absorbance changes of methylene blue incubated with laser irradiation for 0, 2, 4, 6, 8 and 10 min; (B) UV-vis spectroscopy of methylene blue incubated with ZDCIR with laser irradiation for 0, 2, 4, 6, 8 and 10 min.

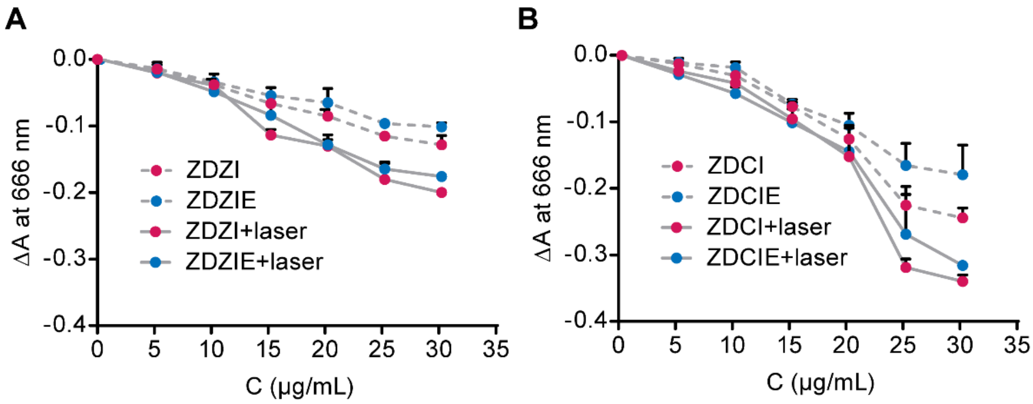

Figure S5. (A) Absorbance changes of methylene blue incubated with different concentration of ZDZI, ZDZIE and (B) ZDCI, ZDCIE with or without laser irradiation for 10 min.

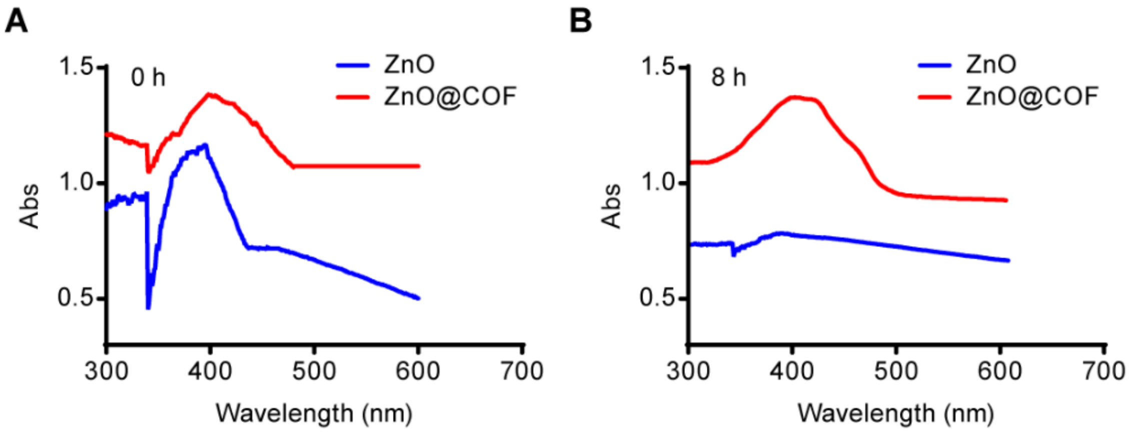

Figure S6. UV-vis absorption band of ZnO and ZnO@COF (200 μg/mL) suspended in deionized water for 0 h (A) and 8 h (B) with NIR for 5 min.

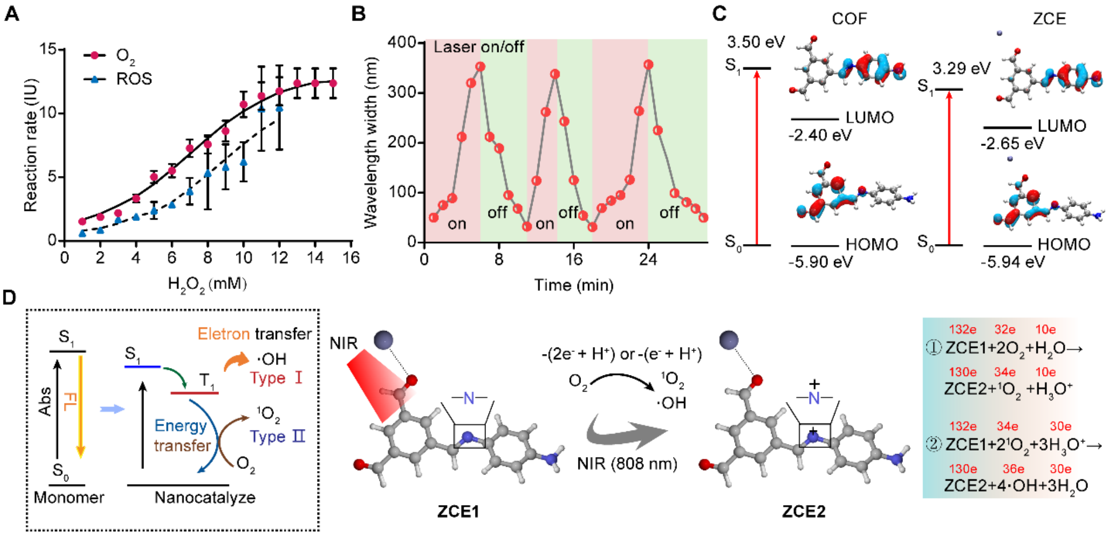

Figure S7. (A) The reaction rate of ZCE to to produce O2 and ROS; (B) The wavelength width variation curves of ZCE over three on (ETS)/off (ground state) cycles under irradiation; (C) Kohn–Sham molecular orbitals of COF and ZCE with orbital energies (eV). (D) Schematic image of ROS generation through both type I and type II mechanisms and inferences on the reaction process based on quantum chemical theory.

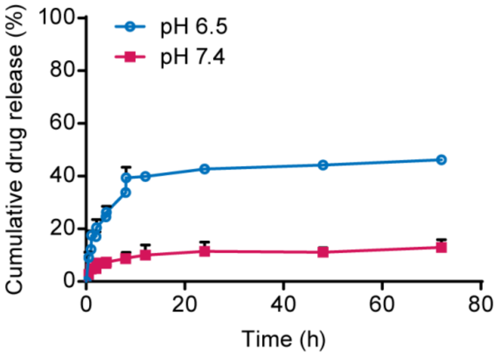

Figure S8. *In vitro* drug release profiles of DOX with laser irradiation for 5 min.

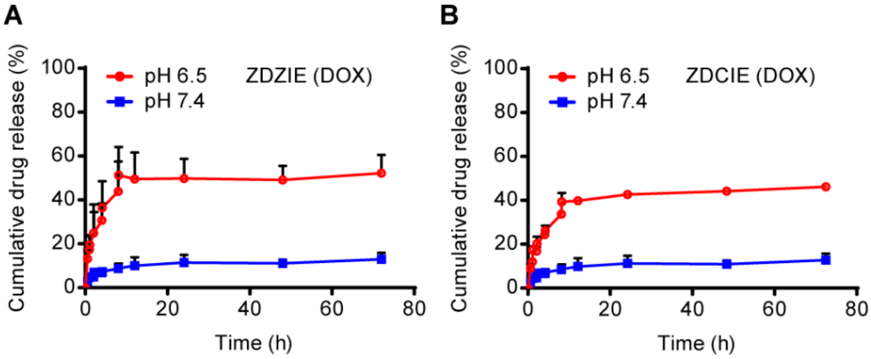

Figure S9. The released profile of different NPs loaded DOX with laser for 5 min.

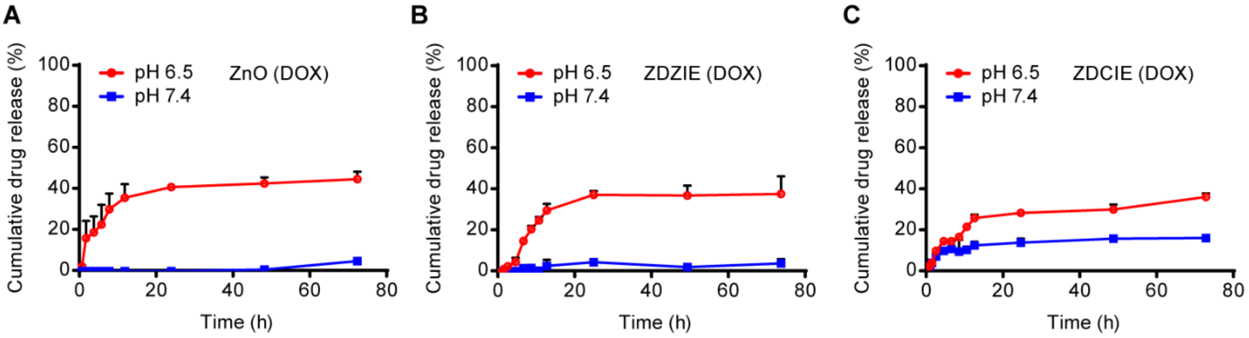

Figure S10. The released profile of different NPs loaded DOX without laser.

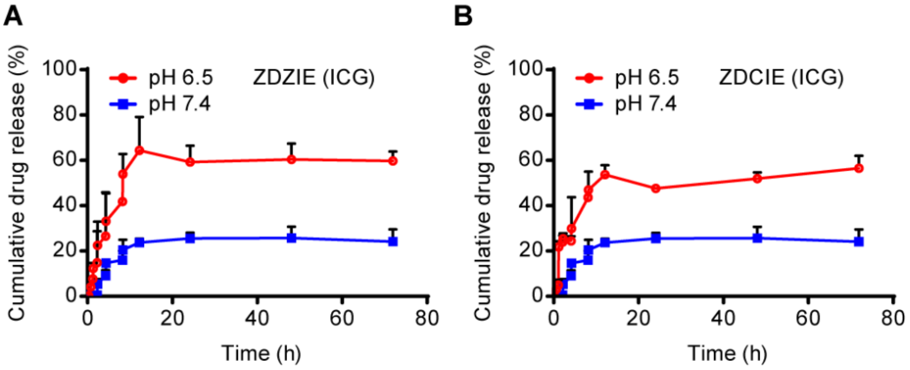

Figure S11. The released profile of different NPs loaded ICG with laser for 5 min.

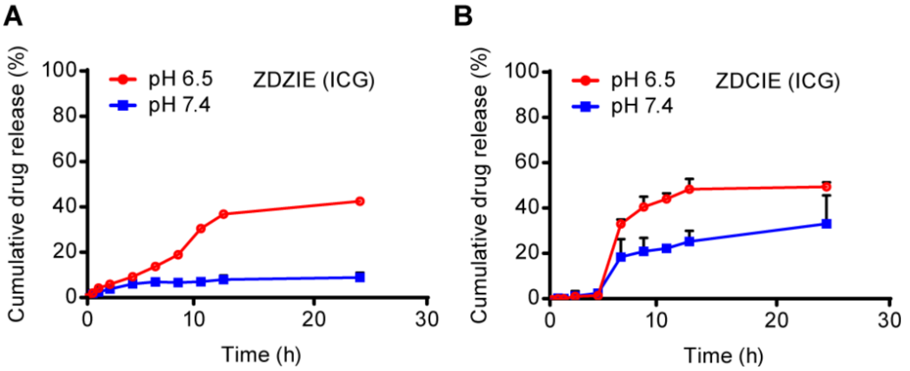

Figure S12. The released profile of different NPs loaded ICG without laser.

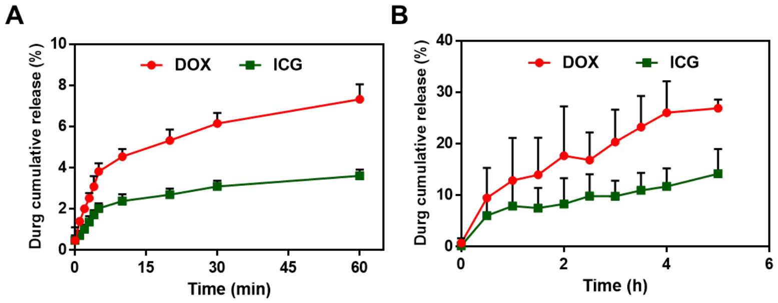

Figure S13. The premature release of the drugs for ZDCIE in the TME (A) and its release efficacy inside 4T1 cells (B).

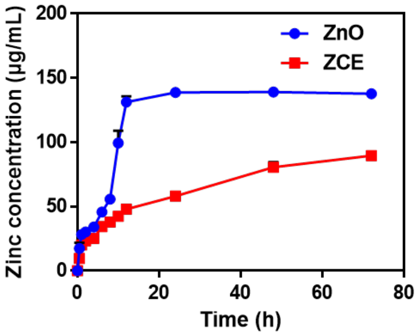

Figure S14. Time-dependent concentration of Zn element of NPs in pH 6.5 medium.

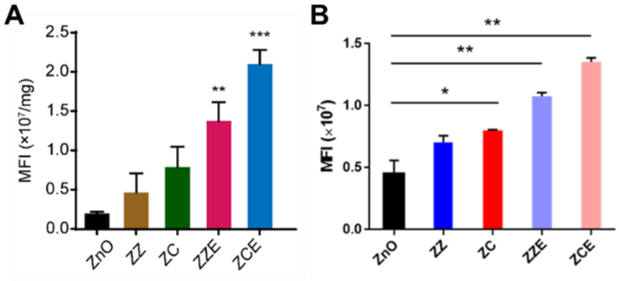

Figure S15. Qualification of the internalized of NPs in 4T1 cells under normal (A) and hypoxia (B) atmosphere. $*p < 0.05$; $**p < 0.01$, and $***p < 0.001$.

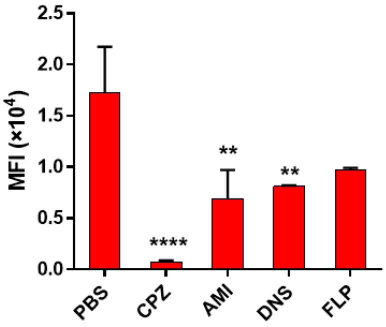

Figure S16. Qualification of the internalized of NPs in 4T1 cells under different inhibitions. $**p < 0.01$, $***p < 0.001$ and $****p < 0.0001$.

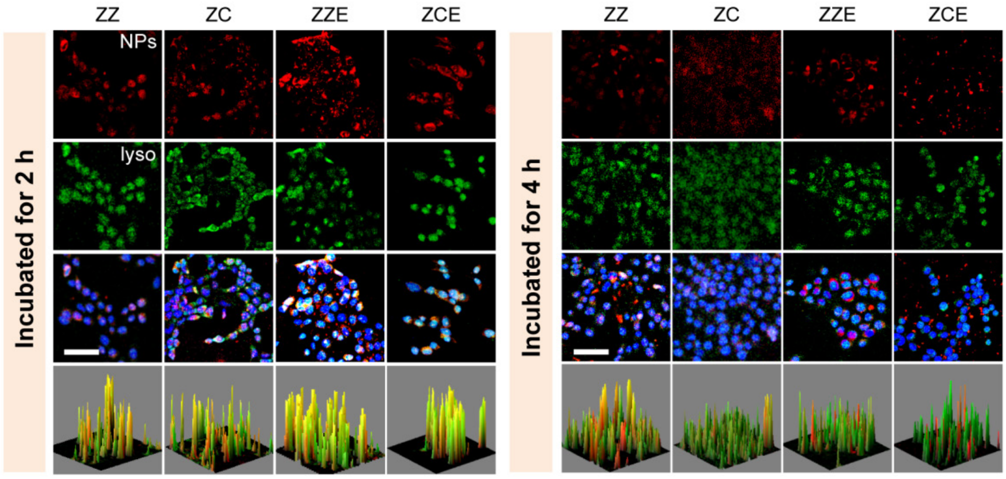

Figure S17. CLSM images of NPs (red) incubated with the lysosome (green) of 4T1 cells for lysosomal escape. Scale bar, 5 μm;

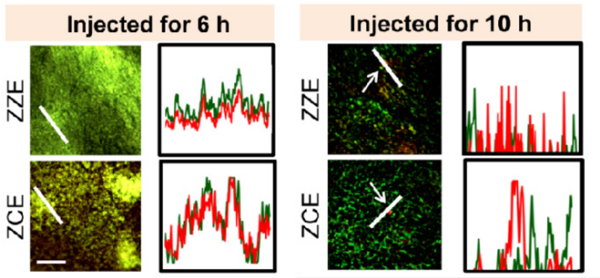

Figure S18. Immunofluorescence staining images of lysosome (green) incubated with RITC-labeled NPs (red) in 4T1 tumor. Scale bar, 200 μm;

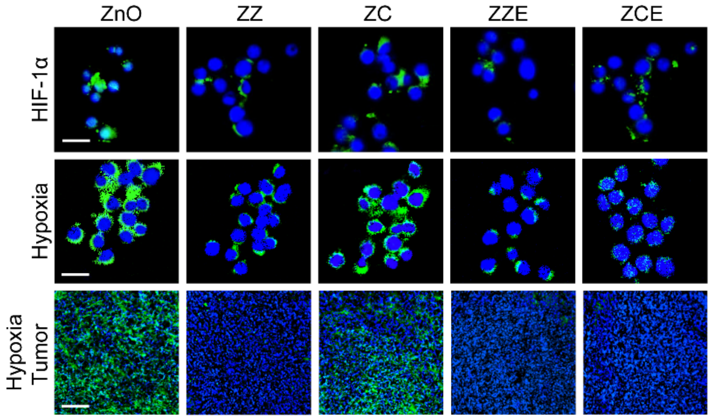

Figure S19. CLSM images of HIF-1α, Pimonidazole (green) and DAPI (blue) in 4T1 cells and tumor tissues incubated with NPs under hypoxic conditions. Cell scale bar, 20 μm. Tumor scale bar, 100 μm.

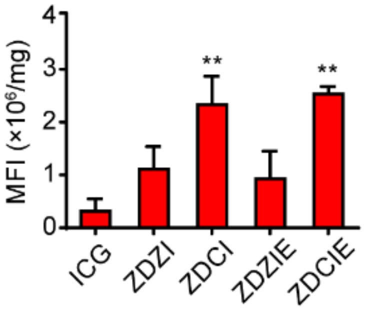

Figure S20. Qualification of ROS detected with DCFH-DA in 4T1 cells incubated with NPs. Compared to ICG group, $^{**}p < 0.01$.

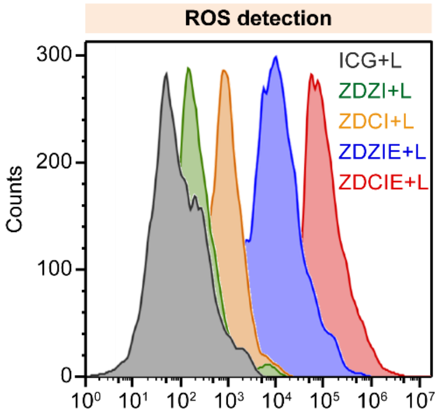

Figure S21. Flow cytometry (FCM) analysis on the ROS production.

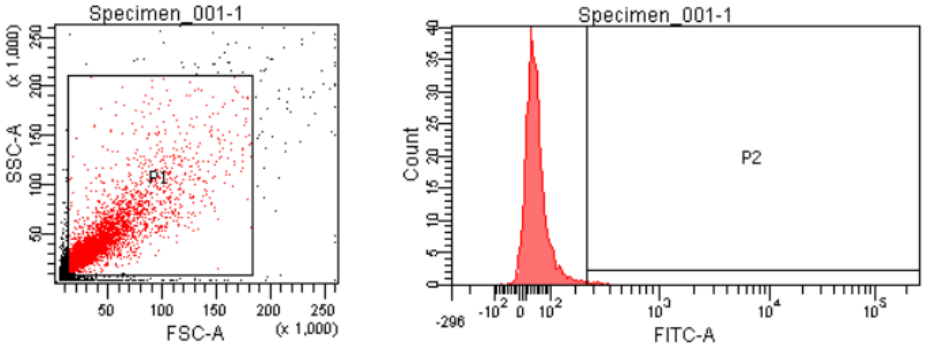

Figure S22. The gating strategy used in the flow cytometry analysis for cell uptake experiments and ROS production.

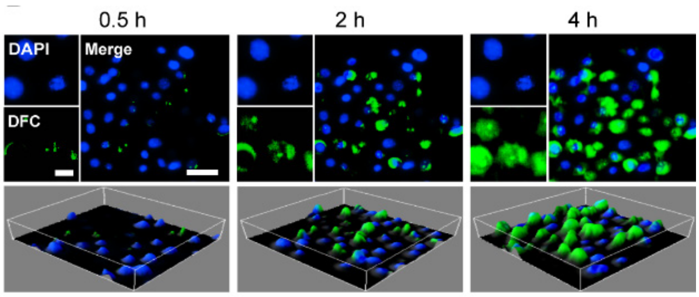

Figure S23. Intracellular ROS detected with DCFH-DA in 4T1 cells incubated with NPs for time series with laser irradiation. Scale bar, 20 μm;

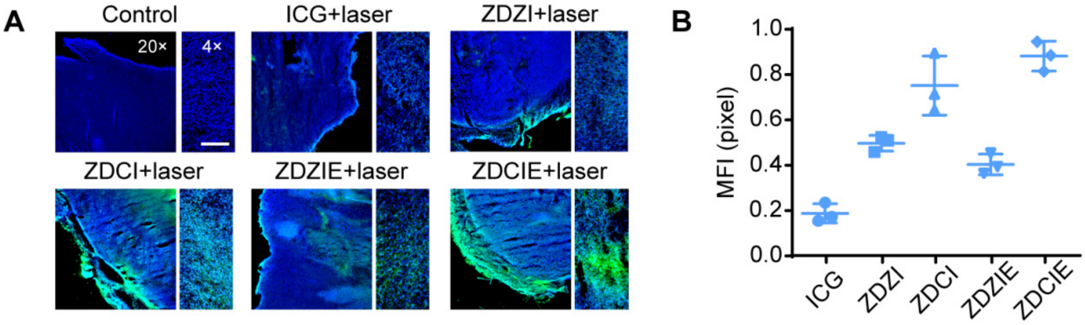

Figure S24. (A) Tumor tissue ROS detected with DCFH-DA incubated with NPs with laser irradiation in tumor tissue; Scale bar, 50 and 100 μm; (B) Qualification of ROS detected with DCFH-DA in tumor tissue incubated with NPs.

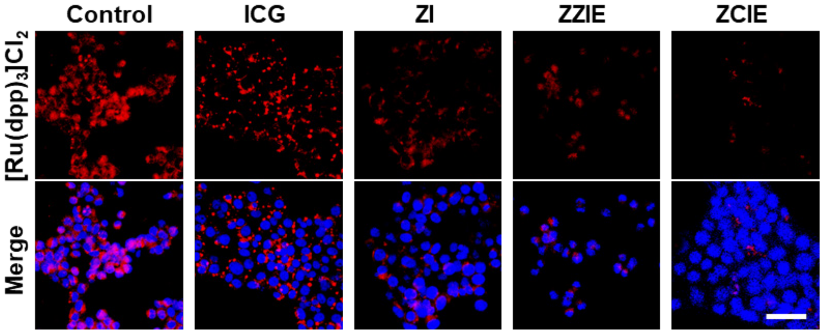

Figure S25. CLSM images of the $^1O_2$ production in 4T1 cells. Blue, nuclei stained with DAPI. Red, $[Ru(dpp)_3]Cl_2$-labeled $^1O_2$. Scale bars, 20 μm.

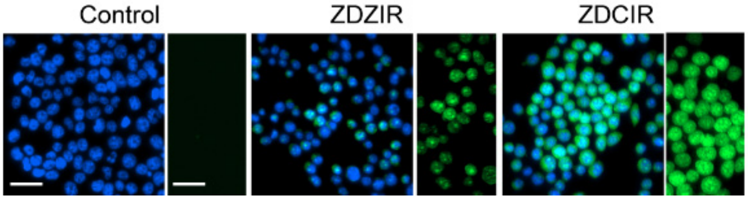

Figure S26. Intracellular CHOP detected in 4T1 cells incubated with NPs for 4 h with laser irradiation. Scale bar, 20 μm;

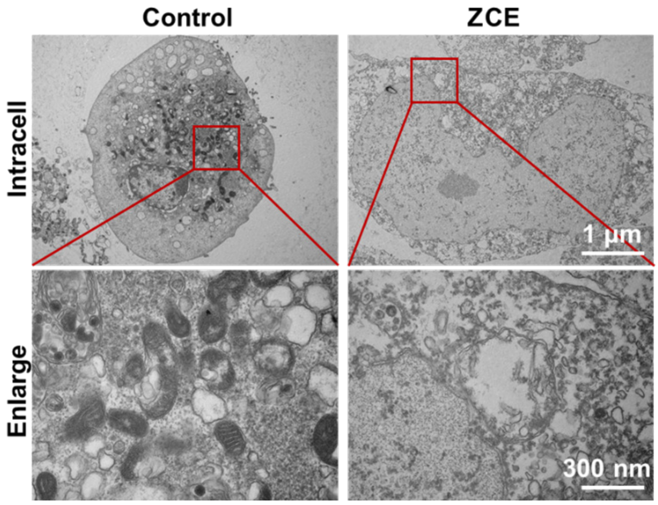

Figure S27. Bio-TEM images of 4T1 cells after inducing Znproptosis.

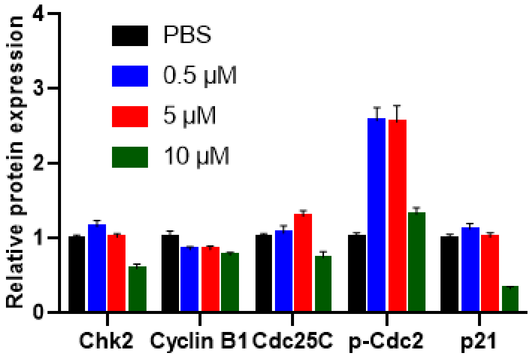

Figure S28. The gray analysis of proteins for cell cycle signal pathways incubated with ZCE in 4T1 cells.

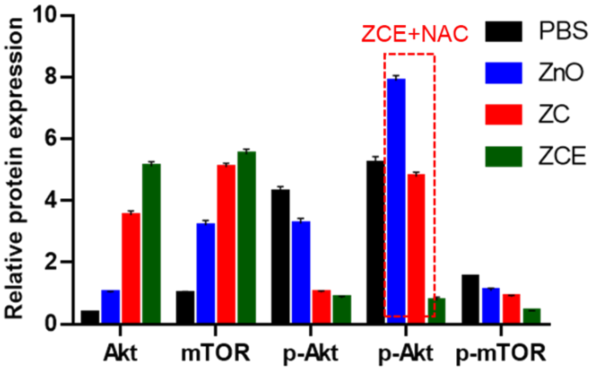

Figure S29. The gray analysis of proteins for Akt/mTOR signal pathways in 4T1 cells.

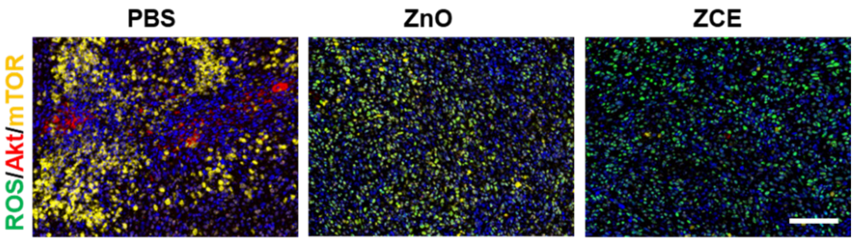

Figure S30. Immunofluorescent staining of ROS (green) and Akt (red) /mTOR (yellow) of tumor tissues after treatment of nanocatalysts. Scale bar, 100 µm.

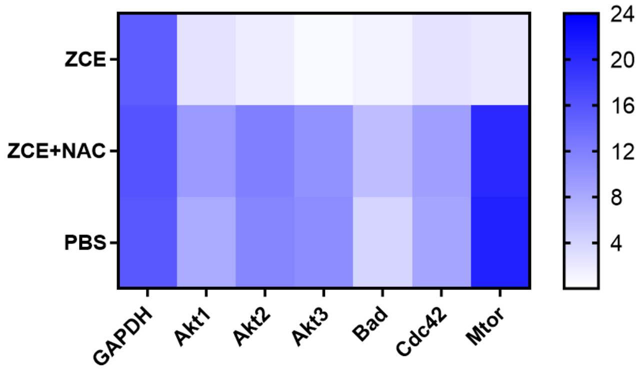

Figure S31. The qPCR array analysis for Akt related genes under NIR for 5 min with/without NAC.

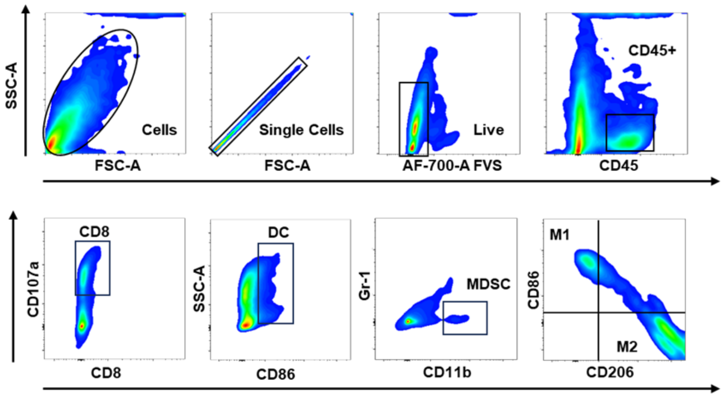

Figure S32. The gating strategy used in the flow cytometry analysis for immune data.

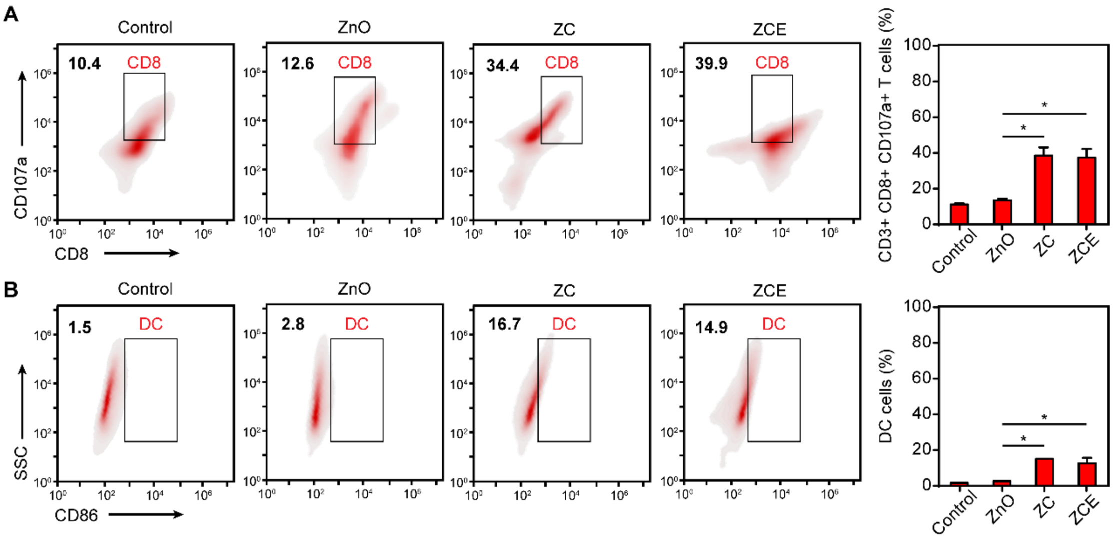

Figure S33. Flow cytometry analysis and corresponding quantification of CD8$^+$ T cells (CD8+CD107a, A) and DC cells (CD86, B) before NIR for 5 min (n = 3, *p < 0.05).

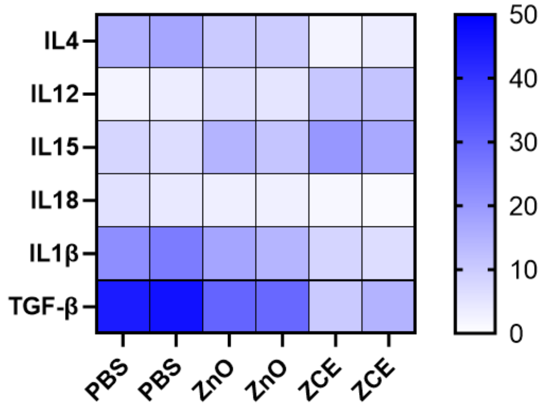

Figure S34. Expression levels of immunity-related cytokines in tumor tissues after different treatments.

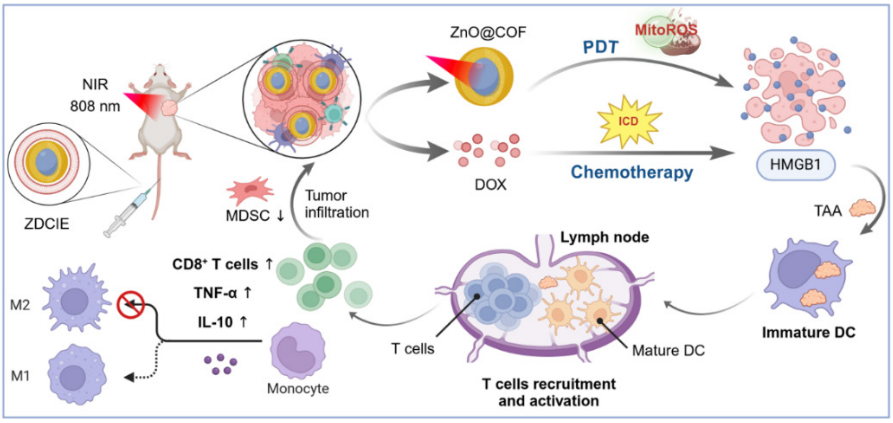

Figure S35. Scheme to describe the mechanism of immunomodulatory effect induced by ZCE.

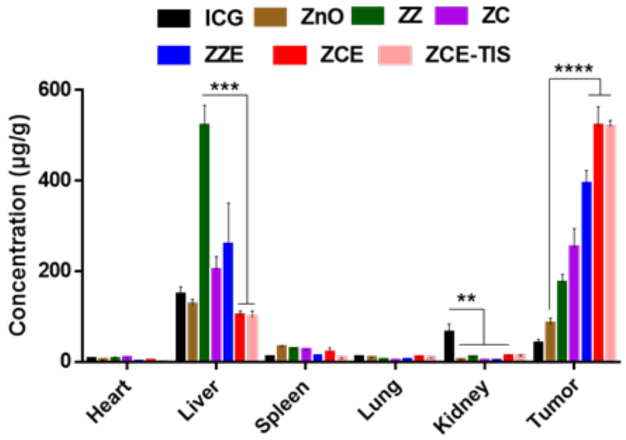

Figure S36. Concentrations of Zn element in main organs including liver at 48 h after injected administration of NPs. All data are presented as the mean ± SD (n = 3). **$p <$ 0.01, ***$p < 0.001$ and ****$p < 0.0001$ by two-tailed Student's t-test.

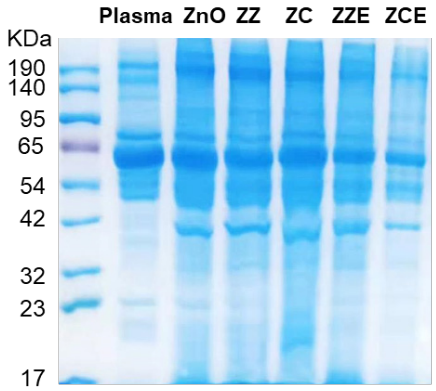

Figure S37. The SDS-PAGE analysis of different NPs after incubated with plasma.

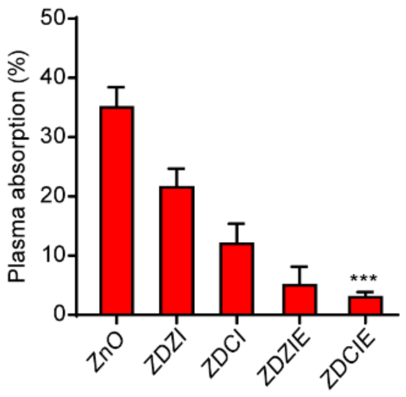

Figure S38. Plasma absorption percentage of NPs after incubated with FBS.

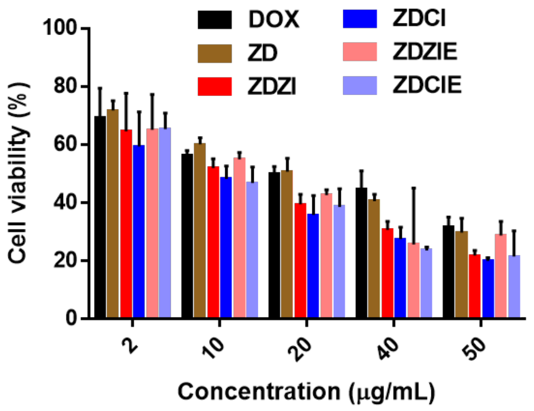

Figure S39. Cell viability in 4T1 cells without 808 nm laser.

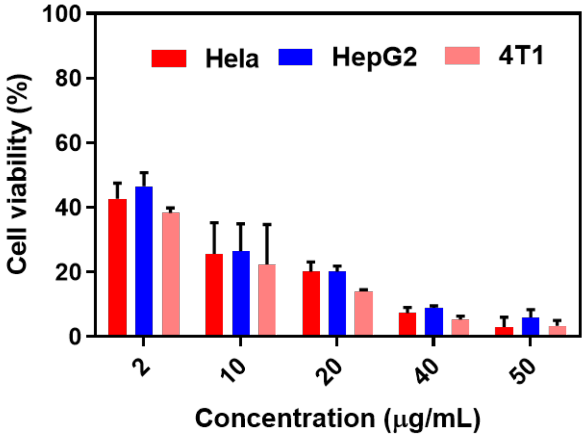

Figure S40. Cell viability of ZDCIE in Hela, HepG-2 and 4T1 cells with laser. The concentrations are the DOX dosage.

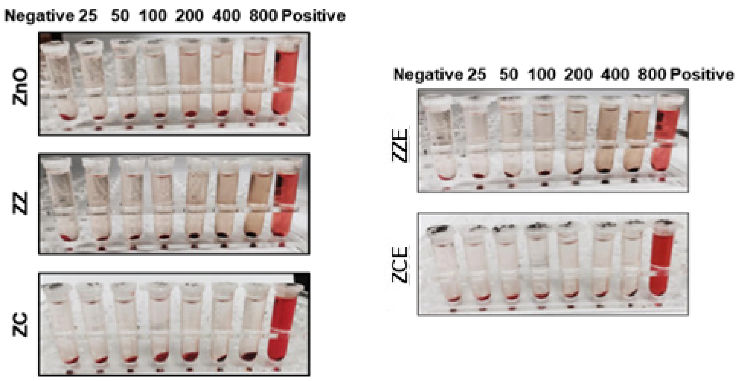

Figure S41. *In vitro* hemolysis condition of different NPs loaded DOX.

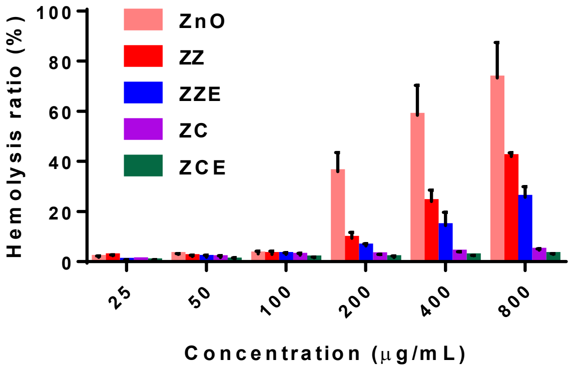

Figure S42. *In vitro* hemolysis ratios of different NPs loaded DOX.

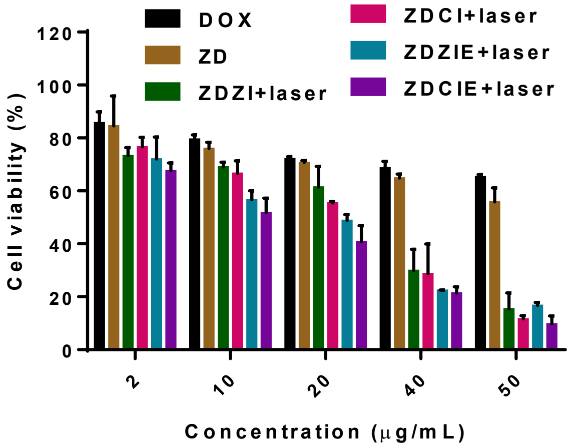

Figure S43. The cell viability of different NPs in the hypoxia atmosphere with laser irradiation.

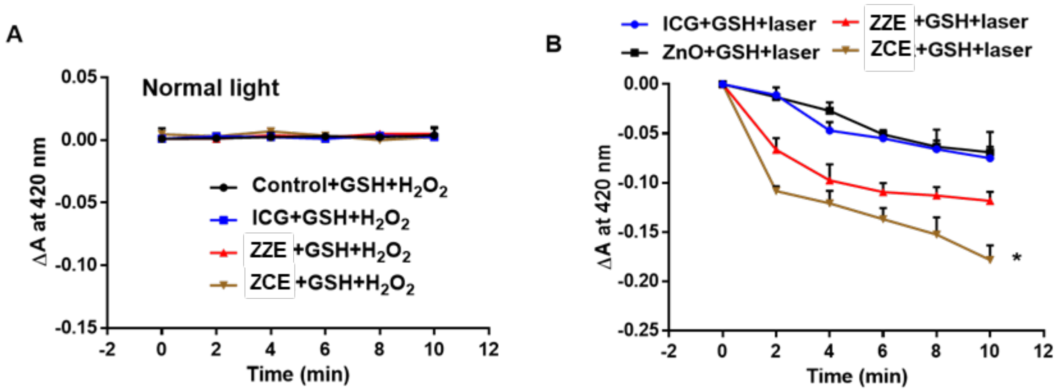

Figure S44. The absorbance changes of DPBF incubated with normal light (A) and laser irradiation (B) for 0, 2, 4, 6, 8 and 10 min incubated with GSH and H_2O_2.

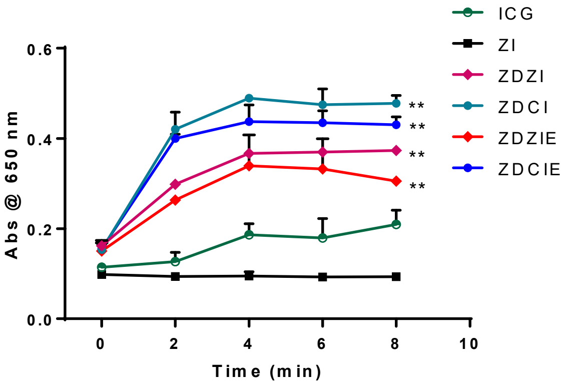

Figure S45. The absorbance changes of TMB incubated with laser irradiation for 0, 2, 4, 6 and 8 min.

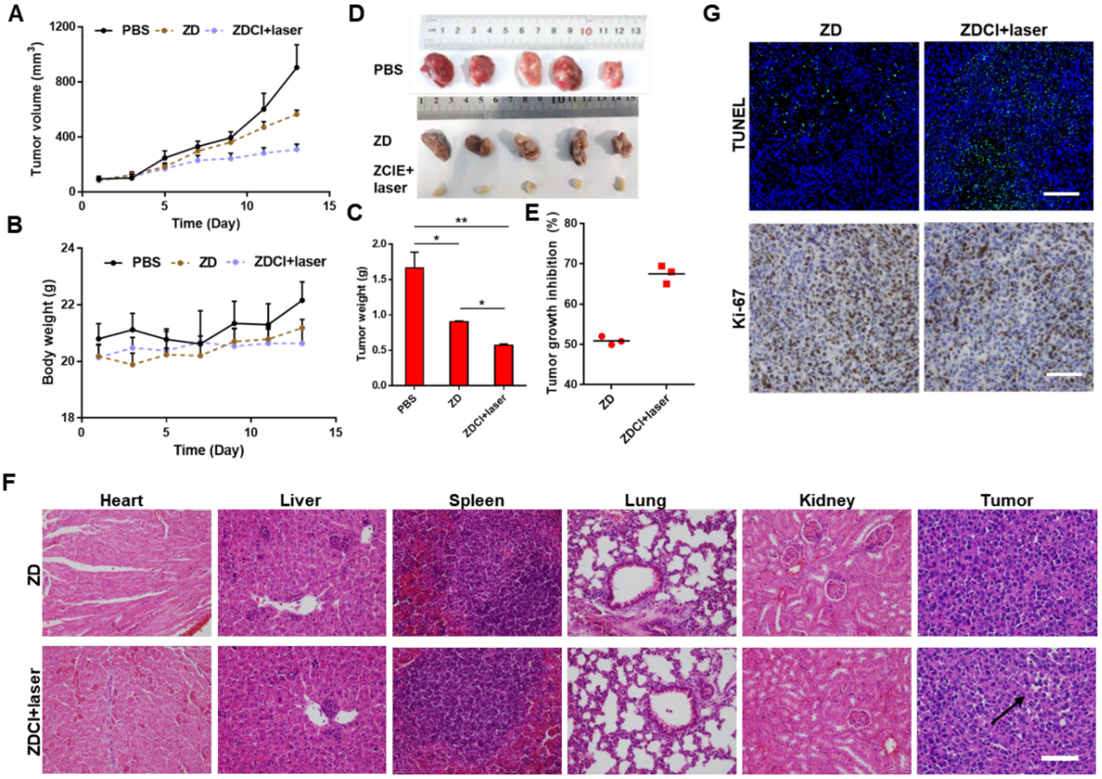

Figure S46. (A) Tumor growth volumes of mice after treatment with nanoparticles examined 14 days after the injection, mean ± SD; (B) Changes in the body weight of the mice after 14-day injection with different NPs via tail veins, mean ± SD; (C) The tumor weight in different groups after 14 days, mean ± SD; (D) Representative tumor images, Scale bar: 4 mm; (E)Tumor growth inhibition rate of mice after treatment with nanoparticles examined 14 days after the injection, mean ± SD; (F)The H&E staining of organs and tumors of the different nanoparticle groups under hypoxic condition. Scale bar: 100 μm. (G) TUNEL and Ki-67 analysis of different treatments under hypoxic condition. Scale bar: 100 μm. ns, $p > 0.05$, *$p < 0.05$, and **$p < 0.01$ (n = 5).

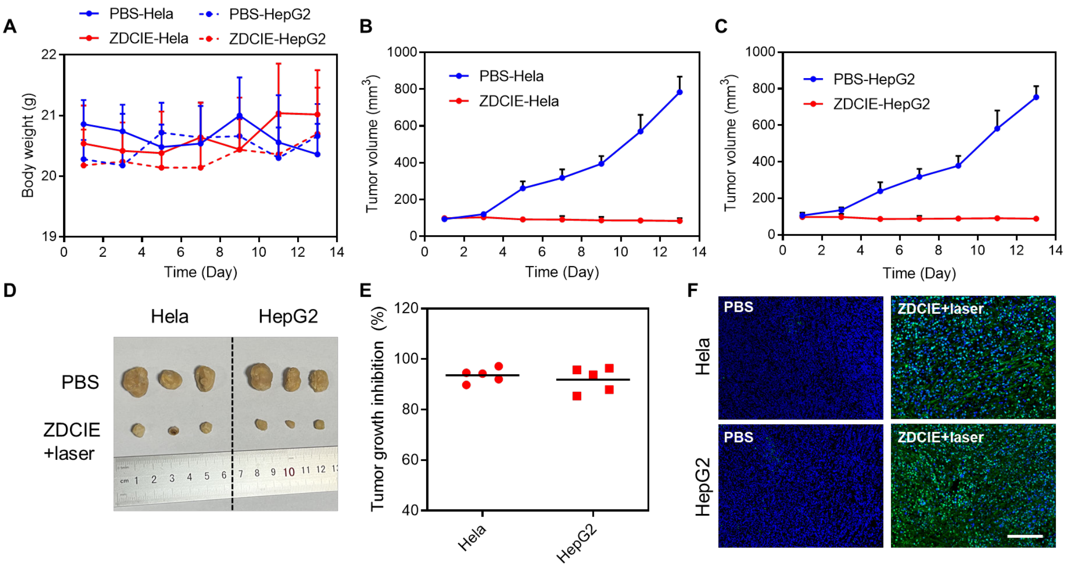

Figure S47. (A) Changes in the body weight of the mice after 14-day injection with different NPs *via* tail veins, mean ± SD; Tumor growth volumes of mice after treatment with nanoparticles examined 14 days after the injection in Hela cells (B) and HepG2 cells (C), mean ± SD (D) Representative images of three superior tumors among five tumors every group, Scale bar: 4 mm; (E)Tumor growth inhibition rate of mice after treatment with nanoparticles examined 14 days after the injection, mean ± SD; (F) TUNEL analysis of different treatments. Scale bar: 20 µm. (n = 5).

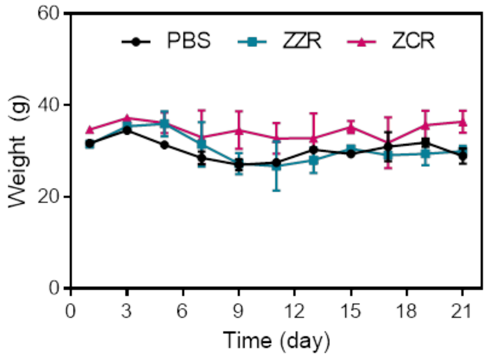

Figure S48. The weight changes of mice incubated with NPs for 21 days.

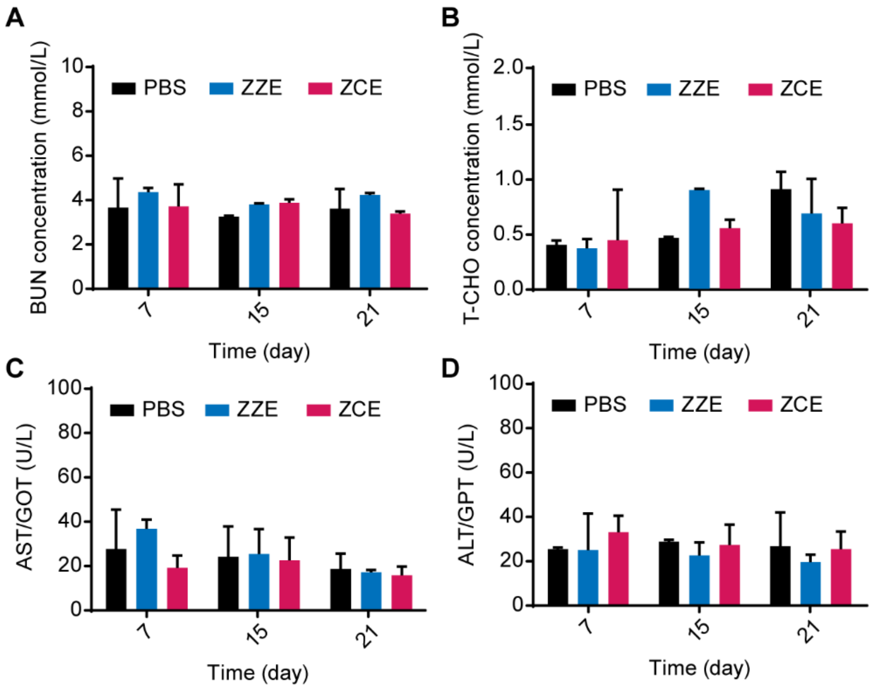

Figure S49. BUN, T-CHO, AST and ALT concentration of samples on day 7, 15 and 21.

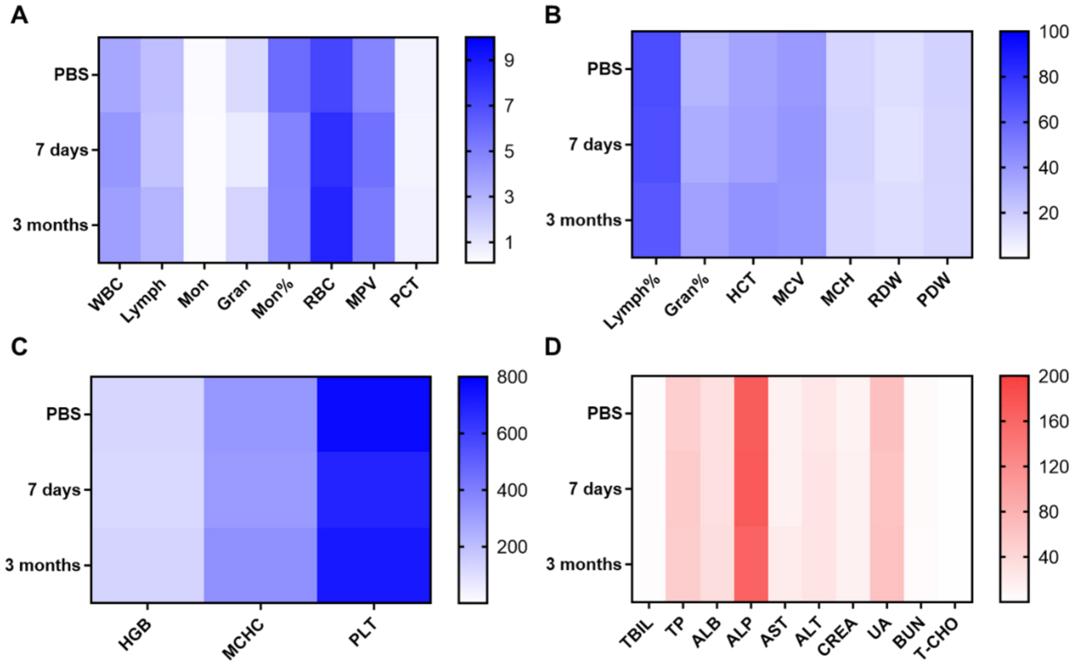

Figure S50. The hematology results (A-C) and biochemical result (D) of mice after treatment with ZCE for 0,7 and 90 days.

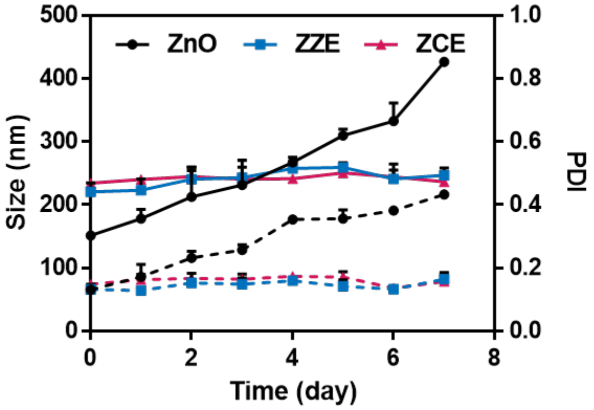

Figure S51. Size distribution and PDI of NPs in FBS at different time points.
